# Trade and Resource Sustainability with Asset Markets

**DOI:** 10.1007/s13235-021-00400-4

**Published:** 2021-11-02

**Authors:** Larry Karp, Armon Rezai

**Affiliations:** 1 Department of Agricultural and Resource Economics, University of California, Berkeley, Berkeley, USA; 2 Department of Socio-Economics, Vienna University of Economics and Business, Welthandelsplatz 1, 1020 Vienna, Austria; 3 IIASA, Schlossplatz 1, 2361 Laxenburg, Austria

**Keywords:** Endogenous property rights, Trade, Asset pricing, Open-access resource, Policy complements, Overlapping generations, Sequential game, Markov perfection, F1, E24, H23, Q28, Q56

## Abstract

Trade changes incentives to protect an open-access natural resource independently of its effect on the resource price. General equilibrium linkages cause resource policy to affect the price of privately owned assets regardless of whether they are used in the resource sector. In the closed economy, the asset market in our overlapping generations setting creates incentives for currently living agents to protect the natural resource. The interplay of the asset market and general equilibrium effects causes trade to reverse these incentives. Trade liberalization and the establishment of formal property rights are policy complements: the former makes the latter more important.

**Supplementary Information:**

The online version contains supplementary material available at 10.1007/s13235-021-00400-4.

## Introduction

Trade liberalization in resource-rich countries with weak formal property rights alters both the incentives and the constraints of common property management. For countries with a comparative advantage in the resource sector, trade increases the resource good’s relative price, increasing the value of protecting the resource but also increasing the temptation to exploit it. Previous literature on the effect of trade in such resource-rich economies emphasizes the effects of this trade-induced change in the * level* of the resource price [9, 15].

We present a novel perspective showing how, for a small country, trade can alter the incentives to protect the resource independently of changes to the current price level. Trade liberalization renders a previously endogenous price exogenous, having subtle effects on the price of a privately owned asset. In our setting, trade reduces the incentives to protect a natural resource, weakening equilibrium protection and possibly lowering welfare. Trade liberalization makes the establishment of property rights more important for preserving resource stocks. In this sense, trade liberalization and the establishment of formal property rights are policy complements.

Three empirical correlations motivate our modeling approach: (i) trade liberalization in small resource-rich countries with weak property rights is associated with increased degradation of resource stocks; (ii) environmental protection and even property rights regimes respond to trade; and (iii) asset prices respond to environmental and policy changes.

The standard trade-and-resource model starts from the premise that imperfect property rights to a sector-specific resource, e.g., fish or forests, attract too many mobile factors to the resource sector even in the absence of trade. Small resource-rich countries with weak property rights therefore tend to have a low autarchic price for the resource good. If such a country liberalizes trade, its domestic price for that good rises, attracting still more mobile factors to the sector, exacerbating the market failure and possibly lowering welfare even in the absence of changes in the resource stock [13]. Reductions in future resource stocks, caused by higher current harvest, aggravate this welfare cost [7, 8, 19, 29].

Hotte et al. [22] and Copeland and Taylor [16] show how trade affects equilibrium resource extraction via the endogenous enforcement of property rights. In their setting, an infinitely lived agent (ILA) with de jure property rights to the resource decides how much to spend to protect the resource from poachers. Trade raises the resource price, increasing the benefit of protecting the owners’ rights, but also attracting poachers, increasing monitoring and enforcement costs. The endogeneity of the enforcement of de jure rights creates another conduit for trade to change resource use and welfare. These papers consider only steady-state effects when current policy makers can commit to future policies.

We also study the effect of trade on equilibrium resource policies. However, current decision makers in our setting cannot commit to future policies, and they do not care about future generations’ welfare. We emphasize a Markov perfect equilibrium; to aid intuition, we also briefly consider the simpler open-loop (but time consistent) outcome. We study the trajectory of policies and resource stocks, not merely the steady state.

In the standard trade-and-resource model, current resource policy affects subsequent resource stocks and welfare. To the extent that there are property rights to the resource, current owners internalize scarcity effects. Previous papers have no assets apart from the resource stock. We assume, in contrast, that the resource sector is open access and we include a separate asset with perfect property rights, outside the resource sector. Without property rights to the resource, there is no market to *directly* reward current agents for conserving the resource.^1^ However, the market for the nonresource asset provides indirect incentives; the resource stock affects labor allocation, thereby affecting the return to this asset. Current policy changes the future resource stock, thus changing future returns to this capital. Because the current asset price incorporates future returns, current owners care about future resource stocks and therefore have an incentive to manipulate them. To isolate this asset price incentive from altruism, we replace the ILA with overlapping generations (OLG) who do not care about their successors.^2^ The incidence of taxes differs across trade regimes [39, 40].^3^ To the best of our knowledge, ours is the first paper to examine how this shift in incidence alters the indirect benefits of conservation, operating through asset markets. In the closed economy, the endogeneity of commodity prices causes self-interested asset owners to protect an open-access resource. Trade, with exogenous commodity prices, reverses incentives, and causes these agents to speed resource degradation.

In either trade regime, the establishment of property rights gives current agents an incentive to protect the resource. The absence of those rights has greater consequences for the open economy compared to the closed economy. Institutional changes such as the creation of private property rights may cause socially costly dislocations. Trade strengthens the case for incurring the social costs needed to establish property rights: trade liberalization and the establishment of property rights are *policy complements*.

A comparison of the hilly regions of Nepal and the flatlands of Malawi illustrates the importance of price endogeneity, which forms the basis for policy complementarity between trade liberalization and the establishment of formal property rights. The poor transportation network in Nepal limits access to external sources of energy and soil nutrients, making inhabitants vulnerable to degradation of forest resources. Inhabitants therefore have a strong incentive to protect these resources. The Miombo woodlands in Malawi are located in flat areas with roads providing access to markets. Otsuka and Place ([43], chap. 10) ascribe the relative lack of success in Malawi common property management in part to the superior market access, which diminishes incentives to protect the resource.

The next section reviews evidence for the three empirical correlations listed above. We then present the model, and analytically establish the results described above. Next, we use numerical methods to establish that the conclusions are robust.

We then discuss several generalizations; our results continue to hold under some of these, but not under others. Our policy conclusions are thus not fragile, but neither are they universal. For example, our assumption that the privately owned asset is fixed (or more generally, that it changes exogenously) means that all of the policy-induced adjustment in the asset market occurs via the price. A more general model with endogenous investment in the privately owned asset would involve both price and quantity adjustment, and would therefore weaken (but not eliminate) the asset price effect that drives our results.

## Empirical Motivation

Data limitations and the associated difficulty of establishing causal relations discourage the use of cross-country econometric analysis, although Ferreira [18] finds that trade is positively correlated with forest degradation where property rights are weak. Most of the evidence linking trade to local resource degradation is based on case studies, chosen for their prima facie evidence of such a link. Therefore, the fact that these studies often find this link does not suggest that trade liberalization typically harms natural resources.

Mammal stocks provide persuasive examples where trade, coupled with weak property rights, harms natural resources. These examples include seals [44], beaver [11], the Arctic Bowhead whale [2], buffalo [46], elephants and rhinos [41]. In all of these cases, the prima facie evidence is compelling, but data limitations complicate the empirical problem.

The trade–resource nexus is also important for forests, fish stocks, and water supplies. In these cases, identifying the role of trade is particularly difficult, because of confounding factors such as increased population pressure. Lopez [37, 38] finds evidence that trade aggravated resource degradation in Ghana and Cote d’Ivoire. Abaza and Jha [1] and Larson and Nash [33] each synthesize six case studies, involving different countries in Africa, Asia, and South America. These 12 case studies demonstrate the complexity and richness of the trade–resource nexus. In some cases, e.g., in Argentina and Senegal, trade and investment liberalization contributed directly to overharvesting of fish stocks. Here, an additional distortion, EU subsidies to EU fleets, compounded the problem of weak domestic property rights. Other examples show why there is not a simple relation between trade and resource use. An EU policy to stimulate livestock production in Ile de la Reunion led to a temporary surge in maize exports from Madagascar, accelerating deforestation; however, previous import *restrictions* in Madagascar, aimed at increasing domestic production of food, led to even greater deforestation. In regions of China and Vietnam, shrimp farming for the export market contributed to the decline of mangroves. EU biofuel policy contributed to deforestation (to develop palm oil plantations) in Southeast Asia, eliciting calls for EU policy changes and subsequent complaints of unfair practices to the WTO, by palm oil producers [20]. These examples show that trade has complicated effects on natural resources, sometimes benefiting and sometimes harming them.

Besley [4] summarizes evidence that property rights are malleable, and Otsuka and Place [43] provide examples of property rights responding to increased market access. Often, increased trade leads to stronger property rights, but Fenske [17] documents that Igbo groups in Nigeria moved from private to common property rights over palm trees in response to increased palm oil trade. Trade increased the value of the palm trees, and monitoring was cheaper under common property than under individual or family property rights. Monitoring and enforcement costs help to determine the equilibrium form of property rights. Abaza and Jha’s [1] and Larson and Nash’s [33] syntheses document many cases of trade liberalization creating the need for, and then inducing, changes in natural resource management policies.

Recent empirical studies find significant links between environmental policy and stock prices [32], house prices [12], and firm profits [10, 36]. These results and the case studies establish the empirical relevance of the three empirical correlations underlying our model. Section 6 discusses generalizations of our model to reflect the complexity of the empirical evidence.

## Model

We describe the competitive equilibrium in an OLG economy for a small open or closed economy under open access. The economy consists of two sectors: manufacturing, *M*, and a resource sector, *R*. The population and capital stocks are both constant. Agents live for two periods. We normalize the stock of capital and the size of each generation to 1. Labor is mobile across sectors, capital is specific to sector *M*, and the endogenously changing resource stock, $$ x_{t}$$, specific to sector *R*.

The model describes a small developing economy in which property rights to the resource stock are nonexistent, and property rights to capital are perfect. The resource sector might have inherited traditional, nonmarket-based institutions, including weak property rights; the (more modern) manufacturing sector does not have this inheritance. Differences in monitoring and enforcement costs might also explain the difference in de facto property rights. Manufacturing capital may consist of machines in a factory. By building a fence and hiring a few guards it is relatively cheap to protect de jure property rights to the machines. The resource stock consists of trees in a forest and of fish in a lake. Monitoring costs make it virtually impossible to protect whatever de jure property rights might exist for the forests and fish. As a consequence, there are (almost) perfect de facto property rights for machines, whereas the resource is (approximately) open access.

The manufacturing good is the numeraire and the price of the resource good is $p_{t}$. In the small open economy this price is exogenous, and in the closed economy it is endogenous, a function of the resource stock and the resource policy. The representative price-taking manufacturing firm chooses capital, *K*, and labor, $L^{m}$, to solve

$$\begin{aligned} \left. \begin{array}{c} \underset{K_{t},~L_{t}^{m}}{\max }\left[ m(L_{t}^{m},K_{t})-w_{t}~L_{t}^{m}- \pi _{t}~K_{t}\right] \\ \text {with }M_{t}=m(L_{t}^{m},K_{t})=\left[ \beta ~\left( L_{t}^{m}\right) ^{1-\frac{1}{\eta }}+\left( 1-\beta \right) ~K_{t}^{1-\frac{1}{\eta }}\right] ^{\frac{1}{1-\frac{1}{\eta }}}, \end{array} \right. \end{aligned}$$

with $w_{t}$ the wage and $\pi _{t}$ the rental rate for capital. As $\beta \rightarrow 0$ we have a fixed, exogenous supply of the manufacturing good. As $\beta \rightarrow 1$, we obtain the Ricardian structure used in previous papers on trade and the environment. For our closed economy, we obtain the model in Karp and Rezai [28] as a special case, letting $\eta \rightarrow 1$. Here, however, our focus is on the comparison between the open and the closed economy.

The representative price-taking resource firm maximizes profits, using a constant returns to scale (in labor, $L_{t}$) harvest function, $R_{t}=\gamma ~x_{t}L_{t}$. The current resource stock is predetermined; with open access (free entry), resource firms ignore the effect of harvest on future stocks. Equivalently, firms set the shadow value of the resource stock to zero. Even without property rights, society can manage resource use by means of an ad-valorem tax, $\tau _{t}$, on harvest. Given resource price $p_{t}$, and the tax, the resource firm obtains $\left( 1-\tau _{t}\right) p_{t}$ units of revenue per unit of harvest. The firm’s problem is

$$\begin{aligned} \max _{L_{t}}\left[ (1-\tau _{t})p_{t}~\gamma ~x_{t}~-w_{t}\right] L_{t}. \end{aligned}$$

Tax revenue is $T_{t}=\tau _{t}p_{t}\gamma x_{t}L_{t}$. If the tax is negative (a subsidy), $T_{t}<0$. In the interest of simplicity, we assume that young agents receive all of the tax revenue or pay all of the fiscal cost. Our results do not depend on this assumption. (Appendix B.5 of ESM discusses the general case when the sharing of fiscal policy between generations is endogenous.)

Agents’ single period utility function is $$u(c_{R,t},~c_{M,t})=\frac{1}{\mu } c_{R,t}^{^{\alpha }}c_{M,t}^{^{1-\alpha }}$$ with scaling parameter $$\mu =\alpha ^{\alpha }(1-\alpha )^{1-\alpha }$$; they spend a constant share, $$ \alpha $$, on the resource good. With price $$p_{t}$$ and expenditures $$e_{t}$$, the indirect utility function, $$v(e_{t},p_{t})=p_{t}^{-\alpha }e_{t}$$, is linear in expenditures. The old agent owns the manufacturing asset, and the young agent owns one unit of labor. A young worker divides income, $$ w_{t}+T_{t}$$, into current consumption and saving for retirement, achieved by purchase of $$s_{t}\in [0,1]$$ shares of capital at price $$\sigma _{t}$$. A young agent who buys $$s_{t}$$ shares of capital in period *t* spends $$e_{t}^{y}=w_{t}+T_{t}-s_{t}\sigma _{t}$$ on consumption. The old agent spends all her income, obtained from renting for a period and then selling her assets, so her expenditure is $$e_{t}^{o}=s_{t-1}\left( \pi _{t}+s_{t}\sigma _{t}\right) $$. The young agent’s savings problem is

$$\begin{aligned} \max _{s_{t}}p_{t}^{-\alpha }\left( w_{t}+T_{t}-s_{t}\sigma _{t}\right) + \frac{1}{1+\rho }p_{t+1}^{-\alpha }\left( s_{t}\left( \pi _{t+1}+s_{t+1}\sigma _{t+1}\right) \right) , \end{aligned}$$

where $\rho >0$ is the pure rate of time preference. An interior solution requires

1 $$\begin{aligned} p_{t}^{-\alpha }\sigma _{t}=\left( 1+\rho \right) ^{-1}p_{t+1}^{-\alpha }\left( \pi _{t+1}+s_{t+1}\sigma _{t+1}\right) . \end{aligned}$$

Because the supply of the asset is perfectly inelastic, in equilibrium $s_{t}=1~\forall t$.

Current wealth depends on future productivity. To avoid multiplicity of equilibria arising from an incomplete boundary condition, we consider the limit, as $H\rightarrow \infty$, of a model with horizon *H*. Using Eq. (1) and $s_{t}=1$, the price of capital, measured in units of the numeraire *M*, equals

2 $$\begin{aligned} \sigma _{t}=p_{t}^{\alpha }\sum _{i=1}^{H}(1+\rho )^{-i}p_{t+i}^{-\alpha }\pi _{t+i} \forall t<H \text { and } \sigma _{H}=0\text {.} \end{aligned}$$

The summand is the present value of future real returns; the factor $p_{t}^{\alpha }$ converts those returns to nominal units.

Equilibrium welfare for the young and old generations, $$W_{t}^{y}$$ and $$ W_{t}^{o}$$, equals:

3 $$\begin{aligned} W_{t}^{o}=p_{t}^{-\alpha }\left( \pi _{t}+\sigma _{t}\right) \text { and } W_{t}^{y}=p_{t}^{-\alpha }\left( w_{t}+T_{t}\right) . \end{aligned}$$

The first equation holds because the old agent consumes all her income and has no future consumption. The second equation comes from substituting Eq. (1) into the young agent’s maximand. This agent’s welfare equals the present value of utility obtained from wage and tax revenue, and is independent of $\sigma _{t}$. A higher asset price reduces the young agent’s current consumption and current utility; the agent’s utility gain in the next period, arising from the higher consumption made possible by the higher next-period asset price, exactly offsets the current utility loss. The old agent’s welfare equals her current utility; the young agent’s welfare equals the discounted sum of her current and next-period utility.

The static equilibrium depends on the resource stock, the asset price, and the tax: $$x_{t}$$, $$\sigma _{t}$$ and $$\tau _{t}$$. We consider two trade regimes: the open economy (free trade) and the closed economy (autarchy). We assume $$0<\beta <1$$ and $$\infty>\eta >0$$, so rents are positive in sector *M* and capital is fully employed; even under trade, the economy never specializes in the resource sector. The two factors earn their value of marginal product: $$\pi _{t}=m_{K}(L_{t}^{m},1)$$ and $$ w_{t}=m_{L^{m}}(L_{t}^{m},1)$$. Full employment of labor requires $$ L_{t}^{m}+L_{t}=1$$. Under trade, for a sufficiently low resource price the economy specializes in sector *M*; in that case, $$ L_{t}=0$$. If both sectors operate ($$L_{t}>0$$), labor arbitrage requires $$ w_{t}=(1-\tau _{t})p_{t}\gamma x_{t}$$. In an open economy, trade is balanced; in the closed economy, domestically produced supply equals domestic demand. The constant expenditure share under Cobb–Douglas preferences implies that the price is $$p_{t}=\frac{M_{t}}{R_{t}}\frac{\alpha }{1-\alpha }$$ in the closed economy; this price is proportional to relative supply, a function of $$x_{t}$$ and $$\tau _{t}$$. Appendix B.4 of ESM provides details of the static equilibrium.

The natural resource stock obeys a logistic growth function:

4 $$\begin{aligned} \left. \begin{array}{c} x_{t+1}=x_{t}+rx_{t}\left( 1-\frac{x_{t}}{C}\right) -L\gamma x_{t}=\left( 1+r\left( 1-\frac{x_{t}}{C}\right) -L\gamma \right) x_{t} \\ =\left( 1+\bar{r}(x_{t},\tau _{t})\right) x_{t};\text { with }\bar{r}(\cdot )\equiv r\left( 1-\frac{x_{t}}{C}\right) -L(x_{t},\tau _{t})\gamma . \end{array} \right. \end{aligned}$$

The intrinsic growth rate is *r*, the function $\bar{r}(\cdot )$ is the actual growth rate, the carrying capacity is $0<C\le \infty$, and $L=L(x_{t},\tau _{t})$ is the amount of labor in the resource sector. The logistic growth model is standard in resource economics. As $C\rightarrow \infty$ we obtain a constant growth rate.

Given a sequence of taxes $\left\{ \tau _{t+i}\right\} _{i=0}^{H}$ and the initial resource stock $x_{0}$, a competitive equilibrium is a sequence of static equilibria (where factor and product markets clear and trade is balanced) and sequences of the environmental stock, $\left\{ x_{t+i}\right\} _{i=1}^{H}$, and asset price, $\left\{ \sigma _{t+i}\right\} _{i=0}^{H}$, satisfying Eqs. (2) and (4).

## Policy Incentives

We assume that agents care only about their own welfare, not about their successors. A political bargain between the two living generations enables them to choose the tax to maximize their joint welfare. The resource tax enables them to manage resource use without incurring monitoring or enforcement costs. The policy failure arises because these generations cannot choose future taxes. Those taxes affect their joint welfare because they affect future returns to capital, thus affecting the asset price, $\sigma _{t}$.

Opening a closed economy to trade causes perverse resource policies to replace socially beneficial ones, harming the resource stock and typically lowering welfare. In contrast to previous models, the effect of the trade regime on equilibrium policies does not depend on whether the world resource price is above the domestic level.

In a closed economy, a small tax decreases the real returns to both capital and labor; in the open economy, in contrast, a small tax increases the real return to capital and decreases the real return to labor. In addition, a tax has opposite effects on the asset price in the two regimes, via the tax-induced change in the resource stock trajectory. Under assumptions described below, we show that in every period the equilibrium policy is a resource tax in the closed economy, and a subsidy in the open economy. The next section drops those assumptions and uses numerical methods to show that the qualitative results are robust.

We present the comparative statics of changes in the tax and the resource stock (Lemma 1 and Proposition 1), and then discuss equilibrium tax policy. Real factor returns, $p^{-\alpha }w$ or $p^{-\alpha }\pi$, equal the amount of utility an agent obtains by renting one unit of labor or capital. National income is $Y_{t}\equiv \left( w_{t}+\pi _{t}+T_{t}\right)$, payments to factors plus tax revenue; real national income, $p_{t}^{-\alpha }Y_{t}$, equals the utility value of national income.

### Lemma 1

In a closed or a diversified open economy: (i) A zero resource tax maximizes current aggregate utility. (ii) A higher tax decreases *L*, the labor in sector *R*, increasing the next-period resource stock.

“Appendix A” contains proofs. Lemma 1.i is standard, and holds generally in convex economies with homothetic preferences. Part (*ii*) is obvious for an open economy, where the commodity price is fixed. The higher tax decreases the return to working in sector *R*, causing labor to leave the sector and reducing current harvest, thereby increasing the next-period stock. The endogenous commodity price in the closed economy moderates but does not reverse this effect.

### Proposition 1

(Factor and goods price effects) For fixed resource taxes: (i) A higher resource stock increases aggregate utility. (ii) In the diversified open economy, a higher resource stock increases the real return to labor and decreases the real return to capital. (iii) In a closed economy, a higher resource stock increases the real return to both factors.

Proposition 1.i arises because a higher resource stock rotates out the production possibility frontier, increasing the economy’s feasible consumption set. The notable result is that a higher stock has opposite effects on the two real factor returns in an open economy (part *ii*), but increases both real returns in a closed economy (part *iii*).

We can decompose the effect, on real returns, of a higher resource stock into a “factor price effect” and a “goods price effect.” The factor price effect measures the change in real factor returns due to a change in the nominal return, holding fixed the commodity price. The goods price effect measures the changes in the real returns due to a change in the commodity price. In both economies, the higher stock increases labor productivity in the resource sector. At a given commodity price, higher productivity in the resource sector attracts labor to that sector. The reduction in manufacturing labor lowers the marginal productivity of capital and raises the marginal productivity of labor in that sector. Because manufacturing is the numeraire good, the nominal return to capital falls and the nominal wage rises. This factor price effect tends to make labor’s and capital’s interests antagonistic. In a small open economy, with exogenous commodity price, only this effect operates.

In contrast, in a closed economy, a larger resource stock and the resulting increased supply of the resource good lowers its price. We call this the “goods price effect”; it is absent in the open economy. The fall in price discourages workers from entering the resource sector. The proof of Lemma 1 shows that the goods price effect and the factor price effect offset each other in the closed economy, leaving the labor allocation independent of the resource stock. Therefore, the general equilibrium effect of the higher resource stock leaves marginal productivity of both labor and capital in the manufacturing sector—and thus the nominal return to capital and the nominal wage—unchanged. However, the lower resource price raises the real returns, $p^{-\alpha }\pi$ and $p^{-\alpha }w$, of both factors. Thus, in the closed but not the open economy, owners of the two factors tend to have similar interests.

To obtain intuition about the relation between the trade regime and the equilibrium management of the natural resource, we provisionally adopt:

### Assumption 1

(i) (Monotonicity) A decrease in current harvest, and the consequent increase in the next-period stock, also increases all subsequent stocks outside the steady state. (ii) (Open loop) In choosing current policy, agents take future tax levels as given.

Assumption 1.i is a statement about the equilibrium outcome. “Appendix A” provides conditions under which this assumption is satisfied. It clearly holds in the limit as the carrying capacity in the logistic growth function becomes large ($C\rightarrow \infty$), but also holds more generally. Assumption 1.ii, in contrast, is a statement about agents’ beliefs regarding future policies. We refer to this assumption as “open loop” because in an open-loop Nash equilibrium each agent behaves as if other agents’ policies do not respond to a change in the state variable (here, the resource stock). Section 5 shows that our policy conclusions do not rely on Assumption 1; that assumption merely helps to explain the conclusions.

Consistent with their lack of altruism, currently living agents choose the current resource tax to maximize the sum of their lifetime welfare, $W_{t}^{y}+W_{t}^{o}=p_{t}^{-\alpha }Y_{t}+p_{t}^{-\alpha }\sigma _{t}$:^4^

5 $$\begin{aligned} \tau _{t}=\arg \max _{\tau }\left( p_{t}^{-\alpha }Y_{t}+p_{t}^{-\alpha }\sigma _{t}\right) . \end{aligned}$$

The first term in the maximand equals real income, and the second term equals real wealth.

### Lemma 2

(Asset price effect) Under Assumption 1, a higher tax increases real wealth, $p_{t}^{-\alpha }\sigma _{t}$, in the closed economy, and decreases real wealth in the diversified open economy.

Lemma 2 identifies an “asset price effect”: a tax has the opposite effect on wealth in open and closed economies. The proof of this Lemma is intuitive.

In the open and closed economy, a tax increases next-period’s stock, and under Assumption 1 all subsequent stocks, thus lowering future real returns to capital in the diversified open economy and increasing future real returns to capital in the closed economy. Because real wealth, $p_{t}^{-\alpha }\sigma _{t}$, is the present value of future real returns, a higher tax increases real wealth in the closed economy and decreases real wealth in the diversified open economy. With this intermediate result, we have

### Proposition 2

(i) Under Assumption 1, in every stage of the *H*-stage game, except for the final stage, agents use a resource tax in the closed economy and a subsidy in the open economy. (ii) In the final stage of this game, agents set the tax to zero.

The intuition for Proposition 2 is straightforward. For both the diversified open and closed economies, a zero tax maximizes real income. Therefore, the incentive to use a nonzero tax depends entirely on its effect on real wealth, $p_{t}^{-\alpha }\sigma _{t}$. From Lemma 2, a tax lowers real wealth in the open economy and increases real wealth in the closed economy. In both economies, real income and real wealth are bounded functions of the tax. Therefore, regardless of future policies, a positive tax increases current aggregate welfare in the closed economy, and a positive subsidy increases current aggregate welfare in the open economy.

*Welfare* Three forces determine the welfare effect of moving from one policy scenario to another, e.g., going from zero taxes to equilibrium taxes in the closed economy; or from equilibrium policies in the closed versus the open economies. First, current taxes, which change with the policy scenario, have a direct welfare effect (conditional on the current stock and future policies); second, future policies alter the asset value, $\sigma$; and third, past policies change the current resource stock. In addition, the welfare effects differ for the young and the old generations. Thus, there are a large number of potential welfare comparisons, each of which involves complicated forces.

Section 5 therefore uses a numerical model, both to calculate equilibrium policies and to discuss some of the most interesting welfare comparisons.

*Policy complements* Based on the results above, we conclude that trade liberalization makes the establishment of property rights more important for preserving resource stocks. In this sense, trade liberalization and the establishment of formal property rights are policy complements.

Our explanation turns on the equilibrium price–marginal cost wedge under an institutional regime, which we denote as $g^{j,k}_t \equiv \left( p^{j,k}_{t}-\frac{w^{j,k}_{t}}{\gamma x_{t}}\right)$. The superscript $j \in \{a,\ f \}$ (for autarchy and free trade) identifies the trade regime, and the superscript $k \in \{oa, \ pr \}$ (for open access and perfect property rights) identifies the property rights regime. At this level of generality, we can say nothing about the magnitude of the equilibrium wedges, so we use only their signs to characterize the policies.

With property rights, denote the private resource rent, or shadow value, as $\lambda ^j_t$ in trade regime *j*. This shadow value equals the present discounted stream of additional (after tax) profits due to one additional unit of the resource, taking the trajectory of resource prices and taxes as given. The shadow value is nonnegative, and strictly positive if the resource is scarce. Resource owners sell their remaining resource *stock* to their successors. These price-taking owners sell the current *flow* up to the point where their after-tax price minus marginal cost markup equals the private shadow value of the resource, $\lambda ^j_t$. With free trade, perfect property rights, and no policy intervention, the efficient wedge (*) is $g^{*}_t =\lambda _t^*>0$.

Under open access, where after-tax profits in the resource sector are zero in both trade regimes, $g^{j,oa} = \tau ^{j,oa} p^{j,oa}$ (suppressing the time subscript). Proposition 2 establishes that $\tau ^{a,oa}> 0 >\tau ^{f,oa}$, so $g^{a,oa}> 0 >g^{f,oa}$. Under open access, trade liberalization changes the sign of the wedge from positive (as in the efficient equilibrium) to negative.

We now consider currently living agents’ incentives to tax the resource when property rights are perfect. Denote $V(x')$ as the present discounted value of the resource firm after current sales, given the remaining resource stock $x'$. Modifying Eq.  5 to include the value of the resource firm, currently living selfish agents choose a resource tax to maximize their current real income plus wealth, $p^{-\alpha }(Y+\sigma (x')+V(x'))$.

Under free trade, the commodity price trajectory is exogenous. The price-taking resource firm (with property rights) is correct in taking these prices as given; agents have no possibility of exercising monopoly power. The firm solves the planner’s problem, so the currently living selfish agents’ equilibrium tax is zero. Therefore, under free trade and property rights, the equilibrium price–marginal cost wedge equals the efficient wedge, equal to the private shadow value of the resource: $g^{f,pr} = g^* =\lambda ^{f}>0$.

In the closed economy with perfect property rights, the equilibrium policy is typically not equal to zero.^5^ We explain why the current generations’ marginal valuation of an additional unit of resource tends to be less than the private shadow value. We then discuss the implications of this conclusion.

An example helps to make our point. Suppose that the resource is nonrenewable, marginal extraction costs are stock-independent and constant, and everyone knows that a new technology will make the resource worthless after *T* periods. Moreover, the stock is large enough to sustain production to the point where price = marginal cost for *T* periods. In this case, the untaxed resource has no value: $V(x')=0=\lambda$. If the current generations could freely dispose of a portion of the stock, they would benefit by creating future scarcity, thereby increasing the value of the resource firm from zero to a positive level. This example illustrates the general possibility that the firm’s private shadow value of the resource (here, zero) is greater than current generations’ marginal valuation of the resource (here, negative).

In a more general setting, extracting a bit more in the current period leads to a smaller stock in the next period. Under Assumption 1.i (monotonicity), the lower next-period stock shifts down the stock trajectory. Taking future policies as given (Assumption 1.ii) the lower future stocks reduce future sales, raising the future price trajectory. The price-taking resource owner ignores this price endogeneity, thereby overstating the marginal value, to current generations, of the resource stock.^6^ Current generations cannot increase the value of the firm by inducing future resource owners to behave as monopolists, but they can achieve a similar result by increasing future scarcity. Moreover, a small subsidy increases current real income, because at the untaxed equilibrium, the price–marginal cost wedge equals the positive resource rent. Increasing current resource consumption increases $Y_t$.

For these reasons, the equilibrium policy under autarchy with property rights might be a resource subsidy.^7^ With this conjecture, endogenous policy undermines the standard benefits to resource conservation arising from property rights in the closed economy. Regardless of whether this conjecture is correct, we know that equilibrium policy in autarchy with open access is a tax. Therefore, the increase in resource conservation arising from property rights is lower in our closed economy OLG model with selfish agents, compared to the more familiar infinitely lived agent model. In contrast, the increase in resource conservation arising from property rights is higher in our open economy OLG model, compared to the familiar model.

In summary, we conclude that establishing property rights likely does more to protect the resource in the open compared to the closed economy. For this reason, we consider trade liberalization and the establishment of property rights to be policy complements.

## Markov Perfect Equilibrium

Assumption 1.ii states that current decision makers ignore the possible effect they have on future policies, operating via changes in the resource stock. In this open-loop setting, policies are time-consistent but not subgame perfect: if an agent deviates, the trajectory of the resource stock departs from the trajectory that agents assumed when choosing their policy. The continuation of the open-loop equilibrium, calculated in the initial period, is not an equilibrium following the deviation. Here we discard Assumption 1, and consider a Markov Perfect Equilibrium (MPE). We solve the problem for a large value of *H* so that the model is approximately stationary. The only directly payoff-relevant state variable is the resource stock, $x_{t}$. Selfish agents in the MPE play a sequential game with their successors, as in Hassler et al. [21], Conde-Ruiz and Galasso [14], and Klein et al. [31].

We continue to assume that the young agent receives all of the tax revenue or pays all of the fiscal cost, and that the current policy maximizes the sum of currently living agents’ lifetime welfare (Eq. 5).^8^ In a MPE, the young agent understands that in the next period, when she is old, policy will be chosen in the same fashion.

A Markov policy function maps $x_{t}$ into $\tau _{t}$. A MPE is a mapping that is the best response, for all $x_{t}$, conditional on the expectation that future policies will be set using this mapping. Using numerical methods, we obtain the MPE and welfare comparisons without Assumption 1.

Denote $\Upsilon \left( x_{t}\right)$ as an *arbitrary* Markov policy, mapping the period-*t* resource stock into the period-*t* tax. If the current stock and tax equal $\left( x_{t},\tau _{t}\right)$, and all future taxes equal $\tau _{t+i}=\Upsilon \left( x_{t+i}\right)$, $i>0$, then $\Upsilon \left( x_{t}\right)$ induces an asset price function, defined recursively:

6 $$\begin{aligned}&p_{t}^{-\alpha }\left( x_{t},\tau _{t}\right) \sigma \left( x_{t},\tau _{t}\right) \nonumber \\&\quad =\left( 1+\rho \right) ^{-1}\left\{ p_{t+1}^{-\alpha }(x_{t+1},\Upsilon \left( x_{t+1}\right) )\left[ \pi \left( x_{t+1},\Upsilon \left( x_{t+1}\right) \right) +\sigma \left( x_{t+1},\Upsilon \left( x_{t+1}\right) \right) \right] \right\} . \end{aligned}$$

Equation (6) restates the equilibrium savings condition, equation (1), highlighting the dependence of the endogenous function $$\sigma \left( x_{t},\tau _{t}\right) $$ on $$ x_{t}, $$ $$\tau _{t}$$, and the function $$\Upsilon \left( x_{t+1}\right) $$.

A MPE is a function $\Upsilon \left( x\right)$ for which: $\Upsilon \left( x\right) =\arg \max _{\tau }W_{t}\left( x,\tau \right)$, with $\sigma \left( x_{t},\tau _{t}\right)$ the solution to equation (6 ), where the next-period tax is evaluated using $\tau _{t+1}=\Upsilon \left( x_{t+1}\right)$. We solve this problem for both the open and closed economies. In both cases, finding $\Upsilon \left( x\right)$ is a standard fixed point problem, which can be solved using the collocation method and Chebyshev polynomials [25, 42].^9^

We also examine two different policy regimes. Under Business as Usual (BAU), the tax is set equal to zero in every period. A Social Planner (SP), in contrast, chooses a tax policy to maximize the present discounted stream of current and future welfare, using agents’ discount factor.^10^

*Calibration* Our baseline calibration sets both $\alpha$, the share of the resource-intensive commodity in the consumption basket, and $\beta$, the wage share in manufacturing, equal to 0.5. Production in manufacturing uses Cobb–Douglas technology, $\eta =1$. We use an annual pure rate of time preference of $1\%/$year, which gives $\rho =0.41$ assuming that one period lasts 35 years. The carrying capacity is $C=1$, so *x* equals the resource capacity rate. We set $r=0.68$, implying an uncongested growth rate of $1.5\%/$year, and we choose $\gamma$ so that the closed economy BAU steady state is $x_{\infty }=0.5$, implying that $\gamma =0.513$. We set the world price at $P=3.377$, so that the open and closed economy steady states are equal under BAU. System (7) collects these baseline parameter values:

7 $$\begin{aligned} \alpha =0.5\text {; }\beta =0.5\text {; }\eta =1\text {; }\rho =0.41\text {;}\ r=0.68\text {; }\gamma =0.513\text {; }P=3.377. \end{aligned}$$

For this parameter set and $x>0.5=x_{\infty }$, production in the open economy is diversified. For $1>x>x_{\infty }$, the endogenous relative price *p* in the BAU closed economy ranges between $1.688<p<3.377$. Thus, for $1>x>x_{\infty }$, opening the BAU closed economy to trade causes an immediate increase in the relative commodity price, as in standard models. However, at the BAU steady state, $x_{\infty }$, the calibration implies that opening the closed BAU economy to trade has no direct effect on current prices. We adopt this calibration assumption to emphasize that *the trade regime changes agents’ incentives to protect the resource in a MPE regardless of whether the trade regime alters the commodity price*. The baseline results we report here are representative of those from a much larger set of parameter values reported in Appendix B.2 of ESM.

### Results for the MPE

This section calculates equilibrium policies in the open and closed economies, under the MPE, BAU, and the social planner

**Fig. 1:**
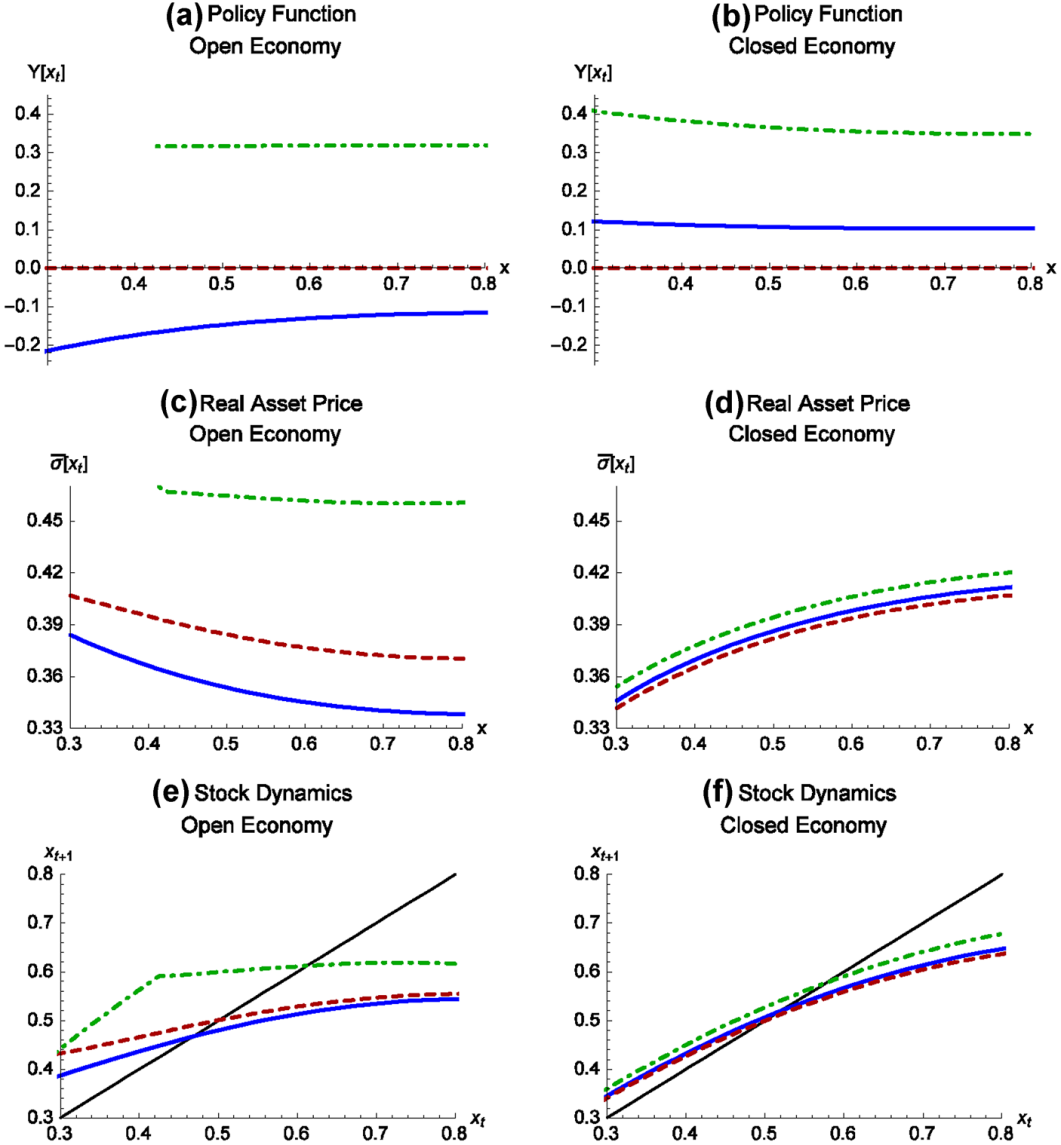
Left panels correspond to open economy and right panels to closed economy. Solid graphs correspond to the MPE, dashed graphs correspond to BAU, and dot-dashed to social planner. The top panels show policy functions, the middle panels show the utility-denominated asset price (real wealth), and the bottom panels show the equilibrium relation between current and next-period stock. Moving from the closed to open economies changes the equilibrium resource tax to a resource subsidy. The equilibrium asset price increases in the resource stock in the closed economy, but decreases in the resource stock in the open economy. Trade lowers the steady-state resource stock

Figure 1 shows results under the MPE (solid), BAU (dashed), and a social planner discussed below (dot-dashed). Left panels correspond to the open economy and right panels correspond to the closed economy. The first row of panels (*a* and *b*) shows the equilibrium policy functions, the second row (panels *c* and *d*) shows equilibrium asset prices, and last row (panels *e* and *f*) shows transition dynamics of the resource stock.

The MPE policy is a tax in the closed economy and a subsidy in the open economy. The closed economy equilibrium tax varies around 10%, and the open economy subsidy varies around 20%, depending on the stock, *x*.

A higher resource stock increases the asset price in the closed economy and decreases the asset price in an open economy (panels *c* and *d*) as in Lemma 2 under Assumption 1. The closed economy MPE asset price is greater than the BAU asset price. In the open economy, agents alive today use a subsidy, lowering the resource stock. If these agents could commit to future taxes, they could raise the asset price and their welfare. This type of commitment is not feasible in a MPE. Agents alive today understand that future agents have an incentive to subsidize resource production. Knowing this, people in the open economy today use a subsidy. Agents face an intergenerational prisoner’s dilemma, where attempts to transfer welfare from the future to the present backfire. The equilibrium subsidy reduces wealth (relative to BAU), reducing young agents’ willingness to pay for the asset.

Panels *e* and *f* show $x_{t+1}$ as a function of $x_{t}$, together with the $45 {{}^\circ }$ line to identify steady states. In the closed economy, the next-period stock is higher in the MPE compared to BAU; trade liberalization reverses this relation. The BAU steady-state stock level equals 0.5 by calibration, regardless of the trade regime. Relative to the BAU level, the steady state increases by $4\%$ in the closed economy MPE and falls by $11\%$ in the open economy MPE. In the closed economy MPE, the domestic consumer price, $p\left( x_{t},\Upsilon \left( x_{t}\right) \right)$, equals the world price at $x_{t}=0.54$. If the economy were to open to trade at this value of *x*, the consumer commodity price remains constant, but the domestic resource tax switches to a subsidy, increasing harvest and causing the stock to fall more rapidly and toward a lower steady state. In previous papers, trade liberalization increases resource use because of differences in domestic and external price levels. In our framework, trade reverses equilibrium policy and increases resource use even in absence of commodity price changes.

**Fig. 2:**
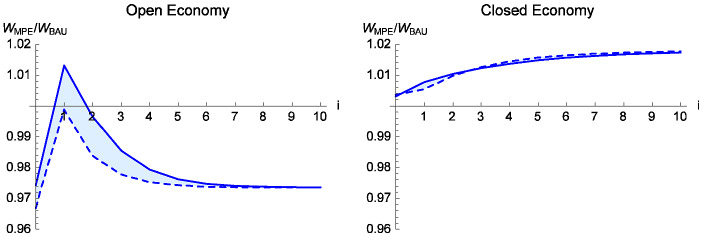
Welfare in MPE relative to BAU with initial resource stock $$ x_{0}=0.5$$ (dashed) and $$x_{0}=0.9$$ (solid) for open economy (left panel) and closed economy (right panel). Period 0 shows combined lifetime welfare of old and young; subsequent periods show lifetime welfare of young. For initial conditions $$0.5<x_{0}<0.9$$, moving from BAU to MPE increases all agents’ welfare in the closed economy; equilibrium policy lowers all agents’ welfare in the open economy, except for those at $$t=1$$ (the first future period)

Figure 2 shows present and future agents’ lifetime welfare under the MPE, relative to BAU levels. For future periods ($$i>0$$), the figure shows the young agent’s lifetime welfare change, and for the initial period ( $$i=0$$) it shows the aggregate lifetime welfare change for the current young and old generations. The dashed curve corresponds to the initial condition $$ x_{0}=0.5$$ and the solid curve corresponds to $$x_{0}=0.9$$. For intermediate initial conditions, the welfare gain lies between these two curves.

In the closed economy, the MPE increases agents’ welfare (the right panel in Fig. 2). Equilibrium resource management increases future stocks, increasing current wealth and future wages. In the open economy, however, agents in period 0 and in every period after period 1 are worse off in the MPE compared to BAU (the left panel). Here, the endogenous policy exacerbates the absence of property rights. If, in the open economy, the initial stock is sufficiently high, the young agent in period 1 has higher welfare in the MPE compared to BAU. This agent has no capital loss; see the comment below equation (3). Due to the high initial condition for *x*, the stock during this agent’s lifetime is still relatively high, so she does not suffer (much) from the subsidy-induced fall in the stock; the subsidy-induced increase in her wage more than offsets the stock-related loss.

Trade liberalization creates the usual static utility gains, but has complicated dynamic welfare effects; these differ under BAU and MPE. Under BAU, at initial stocks above the steady state, the move from the closed to the open economy causes a fall in the asset price (Fig. 1, panels *c* and *d*). Nevertheless, due to the usual static utility gain, trade (under BAU) increases lifetime welfare of agents in the initial period. The lower future stock due to higher harvest reduces agents’ welfare in all subsequent periods, except in the steady state; the BAU steady state, and welfare, is the same in both trade regimes, by calibration.

Trade has a greater welfare effect in the MPE, compared to BAU. The switch from a tax (in the closed economy) to a subsidy (in the open economy) causes a large fall in asset value; except for very high initial stocks, even the generations in the initial period have lower welfare in the open compared to the closed economy. All later generations have lower welfare under trade, even at the steady state, because the economy under trade continues to use a resource subsidy. Appendix 5.3 further discusses the welfare effects of trade liberalization when we hold fixed the policy regime, BAU or MPE.

### Results for the Social Planner

Due to its inherent interest and also to provide a benchmark, we compute the equilibrium to a social planner (SP) with the same pure rate of time preference as individuals. This planner has a standard (time consistent) control problem:

8 $$\begin{aligned} \max _{\left\{ \tau _{t}\right\} _{t=0}^{\infty }}\sum _{t=0}^{\infty }\left( 1+\rho \right) ^{-t}p(x_{t},\tau _{t})^{-\alpha }Y(x_{t},\tau _{t}) \end{aligned}$$

subject to Eq. (4) and an initial condition on the stock. The SP has the same problem as agents with perfect bequest motives and the ILA. Here we summarize the main results, providing more details, and intuition, in Appendix B.3 of ESM.

The dot-dash graphs in Fig. 1 show the equilibrium policy functions, asset prices, and state transitions for the SP. In both the open and closed economies, the SP uses a resource tax, increasing the equilibrium stock and tax trajectories compared to both BAU and MPE. Under the SP, opening a closed economy to trade lowers the steady-state tax but increases the stock. In this sense, trade makes resource protection cheaper.

Because the SP corrects the open-access distortion, opening the economy to trade eliminates the remaining distortion, necessarily increasing the discounted stream of utility. Some of this increased utility appears as capital gains, which the first old generation appropriates. The planner’s objective is to maximize the discounted stream of utility, not, for example, steady-state utility. Thus, even in a standard Ramsey model, there is no presumption that trade, or any other movement from second to first best, increases utility in every period, e.g., in the steady state. In our calibration, except for initial conditions above 0.9, trade lowers all subsequent generations’ welfare under the SP regime.

We also have the following welfare comparisons between the SP and BAU: In the closed economy, the SP lowers first period aggregate utility (as in standard Ramsey models) but increases lifetime aggregate welfare of currently living agents if $x_{0}<0.91$. The current old generation obtains the capital gains due to the SP program, and these exceed the loss that the young generation suffers due to the fall in wages. At higher stocks, the capital gains do not compensate the loss, and the SP lowers current aggregate welfare. The SP increases future agents’ welfare. The SP policy increases initial aggregate welfare in the open economy much more than in the closed economy; the magnitude of the steady-state welfare increase is similar in the two. For high initial stocks, the SP policy lowers welfare of the young born at $t=1$. That generation would not have suffered much from a low stock under BAU, but it has a lower real wage when the SP taxes the resource.

### A Change in the Trade Regime

**Fig. 3:**
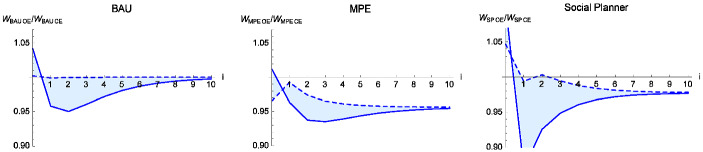
Welfare in the open economy relative to the closed economy under BAU (left), MPE (center) and social planner (right), with the initial resource stock $x_{0}=0.5$ (dashed) and $x_{0}=0.9$ (solid)

Here, we compare the welfare effect of moving from a closed to an open economy, holding fixed the policy environment (BAU, MPE, and the social planner). Figure 3 shows these welfare changes, conditional on BAU, MPE, or the social planner, using the baseline parameters. The horizontal axis (labeled *i*) shows the number of periods from the time at which the closed economy opens to trade. The left panel shows the ratio of open-economy to closed economy welfare under BAU; the middle panel shows this ratio in the MPE, and the right panel shows this ratio under the social planner. As above, the dashed curve shows the ratio given the initial condition $x=0.5$, and the solid curve gives the ratio at initial condition $x=0.9$.

Under BAU, both open and closed economies have the steady states $x=0.5$, so at this value, opening the closed economy has no effect (the welfare ratio equals 1). If the initial stock is high, the initial generations have higher welfare under trade. At stocks above the steady state, the domestic price is below the world market price. The higher price leads to high extraction in the current period and lower ones in subsequent ones, increasing aggregate welfare of currently living agents ($i=0$) and lowering welfare of each future young generation.

In the MPE, all generations are worse off in the open economy, except possibly the first generation if initial stocks are large (Fig. 3, middle panel). The economy reaps the standard static gains from trade, but trade reverses the incentives to protect the resource stock. The lower resource stock lowers future generations’ welfare. If the initial stock is high, then the initial closed economy price is low. In this case, the standard gains from trade may be large enough that trade improves welfare for those alive in the first period. However, for most initial stock levels, and for all future generations, the switch from resource protection to increased exploitation is more important than the standard gains from trade; here, trade lowers welfare.

Under the social planner, opening up to trade puts the economy in a first best world and necessarily increases the present discounted sum of welfare, but need not increase welfare for every generation. The right panel of Fig. 3 shows that trade lowers welfare for most future generations. The trade-induced fall in future generations’ welfare comes from the fact that single period utility is linear in income. With a constant commodity price, the planner has no incentive to smooth consumption. Comparison of panels *c* and *d* of Fig. 1 shows that trade increases the asset price. The old generation in the first period captures all of these capital gains, which in this example exceed 100% of the gains from trade.

**Fig. 4:**
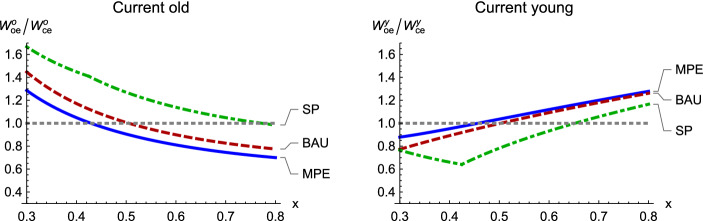
Lifetime welfare of current generations in the open economy (*oe*) relative to the closed economy (*ce*) for the Markov perfect (MPE), business-as-usual (BAU), and social planner (SP) equilibria. Trade lowers the agent’s welfare at stock values where the graph is below the horizontal line at 1

Figure 4 disaggregates the period-0 welfare effect of trade liberalization (from Fig. 3) into welfare changes for the young and the old, for different stocks and policy regimes. The graphs show the ratio of lifetime welfare under open versus closed economies. Trade increases an agent’s welfare in a policy regime if the relevant graph of ratios in Fig. 4 is above 1.

We first discuss the graphs for the young agent, the right panel of Fig. 4. Our simulations use $\eta =1$, where we obtain closed-form expressions for real wages, and thus a closed-form expression for the young agent’s welfare ratio, conditional on the policy (Appendix B.4 of ESM). Using these results, we can show that the elasticity of the ratio under BAU, with respect to the stock, is $1-\alpha >0$. Thus, the slope of the ratio is positive. Provided that the policies are fairly insensitive to the stock (as Fig. 1 shows) these results mean that the young agent’s welfare ratio is also increasing in the stock in the MPE.

Under BAU, the open and closed economy steady states are $x=0.5$ by calibration. At this value, changing the trade regime does not affect either generation’s BAU welfare, so both ratios are 1. Because the BAU welfare ratio for the young agent increases in the stock, the ratio is above 1 if and only if the initial stock is above its BAU steady state. The similarity of the MPE and BAU graphs implies that trade liberalization has similar effects for the young generation in these two policy regimes.

The social planner shuts down the resource sector for low stocks, so over that range the open economy real wage does not depend on the stock. However, the closed economy real wage increases with the stock. Therefore, at low stock levels, an increase in the stock lowers the ratio. At sufficiently high stocks, where the social planner begins to operate the resource sector, the real wage increases with the stock in both trade regimes. However, it increases more quickly in the open than in the closed economy, so the ratio rises. The ratio exceeds 1 only for stocks close to the carrying capacity, $x=1$. In all three policy scenarios, the young agent benefits from trade if and only if the gains from trade are very high, which occurs when the stock is high.

We now turn to the old agent’s welfare ratio, the left panel of Fig. 4. Figure 1 shows that under both MPE and BAU, the real asset value increases with the resource stock in the closed economy and decreases in the open economy. Note again that the MPE policy functions are quite insensitive to the stock. Therefore, the current real return to capital increases with the resource stock in the closed economy, and it decreases in the open economy. Consequently, the ratio (in the open versus closed economies) of the sum of the asset value and the current return falls with the stock.^11^

As noted above, the BAU ratio for the old generation is 1 at $x=0.5$. Because this ratio falls with the stock, it exceeds 1 if and only if $x < 0.5$. The critical resource stock below which the open country exports the manufacturing good is 0.5 under BAU. In all three policy regimes, the country exports the manufacturing good if the resource stock is sufficiently low. It is not surprising that the old generation tends to benefit from trade in circumstances where the country exports the manufacturing good, the commodity where capital is the fixed factor. The BAU and MPE ratios are close together.

Similar forces are at work under the social planner. For very low stocks, the country exports the manufacturing good under trade; liberalization increases the relative price of this good (lowers the price of the resource good), benefiting capital, the fixed factor in the manufacturing sector. Therefore, the welfare ratio is above 1 at low resource stocks. The ratio falls with the stock for (essentially) the same reason as in the other policy scenarios. The ratio falls below 1 (not shown) only for very high stock levels.

## Extensions

Our functional assumptions make the asset price effect transparent, leading to a simple conclusion: opening a closed economy causes perverse resource management policies to replace beneficial policies, harming the environment and reducing welfare for most or all agents. As Sect. 2 emphasizes, the trade–resource nexus is in fact much more complicated and ambiguous. Here we show that our policy conclusions continue to hold for some, but not for all extensions.

*International trade in factors* If commodity trade is associated with international factor mobility, then the international market fixes factor prices, eliminating agents’ incentive to use *any* resource policy under trade. The movement from autarchy to commodity + factor trade changes the equilibrium resource-protecting tax to laissez faire. Trade eliminates but does not reverse the incentives to protect the resource.

*Intersectoral mobility of capital* We follow Hotte et al. [22] and Copeland and Taylor [16] and many other papers in this literature in assuming that labor is the only mobile factor. Karp [26] shows that even in a static setting, allowing capital as well as labor to be mobile across sectors alters the factor price effects of increased resource protection. For example, a higher resource stock could increase the nominal return to both factors, altering the “factor price effect” described above, and creating an incentive to protect the resource even under trade. Thus, this extension could overturn our result.

*Replacing Cobb–Douglas with homothetic utility* Proposition 1 also holds for general homothetic preferences, for a sufficiently large sector-*M* elasticity of substitution, $\eta$. Cobb–Douglas preferences greatly simplify the arguments, and enable us to state the result for all $0<\eta <\infty$, but those preferences are not necessary for the results. However, with homothetic utility and small $\eta$, the factor price effect dominates the goods price effect in the closed economy. In this case, a larger resource stock lowers the real return to capital in the closed economy, eliminating current agents’ incentives to protect the resource there.

*Making indirect utility nonlinear in income* The Cobb–Douglas utility function implies that indirect utility, $V\left( p\right) y$, is linear in income, where $V\left( p\right)$ is a price index and *y* is an agent’s income. This linearity implies that the elasticity of intertemporal substitution is infinite, and leads to the simple expression of the asset price, equation 2. For a monotone increasing function *G*, we can replace indirect utility with $G\left( V\left( p\right) y\right)$. Karp et al. [27] consider this extension in a closed economy one-commodity framework (where $V\left( p\right) =1$). If, for example, $G\left( \cdot \right) =\ln \left( \cdot \right)$, intertemporal income and substitution effects cancel. In the absence of taxes, the young agent saves a constant fraction of her income, equal to her wage. This savings rule creates a linear relation between the wage and the asset price, which is independent of resource stocks. More general (and more interesting) cases lead to more complicated asset price equations, requiring numerical methods.

*Endogenously changing capital stock* We follow *all* previous papers in this literature, in assuming that only the resource stock changes endogenously. If the stock of privately owned capital were also endogenous, its price would still depend on the resource stock, provided that the production possibility frontier between the consumption good(s) and the investment good is strictly concave. Quantity adjustment would reduce but not eliminate the asset price adjustment that drives our results. Therefore, we conjecture that the introduction of trade would have a smaller but qualitatively similar effect on equilibrium policy in a model with endogenous investment.

Introducing endogenous investment into an OLG model with a stock externality requires an additional state variable (the stock of privately owned capital), and it also raises conceptually difficult issues. Karp et al. [27] study this kind of model, but only in a closed economy with a single consumption good. Absent trade in factors, a trade model requires at least two consumption goods. With three production sectors (the investment good and the two consumption goods), a sector-specific model is either not plausible or not useful. If we assume the existence of an exogenous sector-specific input in the investment sector, we recreate in a different sector the problem that the introduction of endogenous capital was designed to overcome. A model that assumes that capital is (for example) used only in the manufacturing and the investment good sector, but not in the resource sector, is arbitrary. Moreover, it would eliminate the simple relation between commodity prices and factor returns, which our analysis depends upon.

*Costly monitoring and enforcement* Monitoring and enforcement (ME) costs are central in some previous papers. We abstract from these costs to emphasize that our results arise even when agents have a first best means of controlling current resource harvests. The distortion occurs because they cannot control future harvests. ME costs in our setting would likely reduce agents’ incentive to use a resource tax in the closed economy. ME costs are less plausible where resource use is being subsidized, so they would likely not alter the incentive to subsidize resource use under trade. Thus, ME costs likely do not change our qualitative conclusions.

*Further extensions* One could imagine many other extensions, e.g., changing the growth function, changing the harvest function (so that there are decreasing returns to labor, conditional on the resource stock), or allowing imperfect property rights to capital as well as the resource stock. Bohn and Deacon [5] show that those imperfections discourage investment (e.g., in oil extraction), and may reduce natural resource extraction.

## Conclusion

In conventional models, if agents have no concern for their successors and no property rights to the natural resource, they have no incentive to use policy to manipulate resource use. Here, opening a closed economy affects the level of resource use if and only if the resource price changes.

Our model overturns both of these predictions. Endogenous asset prices give agents an incentive to use resource policy. Trade alters the link between asset prices and resource policy and thereby changes incentives to protect a natural resource. This change occurs even if liberalization has no immediate effect on commodity prices. The equilibrium policy in our OLG setting is a resource tax in the closed economy and a subsidy in the open economy.

Markets link factor, commodity, and asset prices; these links cause changes in resource policy or the resource stock to alter the prices even of assets not used in the resource sector. Asset owners care about those price effects, regardless of their intrinsic concern for the resource stock or future generations’ welfare. Trade changes the link between resource policy and asset prices because trade renders an endogenous commodity price exogenous. The qualitative effect of a change in resource policy or the resource stock on the *nominal* return to a factor, does not depend on the trade regime. However, the qualitative effect of change in policy or the stock on the *real *return to a factor does depend on the trade regime. In the closed economy, real returns to both capital and labor move in the same direction, following a change in policy or the stock. In contrast, in the open economy the real returns to capital and labor change in opposite directions in response to a policy or stock change.

Self-interested incentives may help to protect resources in a closed economy, even without property rights. Trade can undermine or reverse these incentives, in which case private property rights might be essential for resource protection. For this reason, we consider trade and the establishment of property rights as policy complements.

We also compare the political economy equilibrium to the social optimum. Regardless of the trade regime, the social planner protects the resource. The overlapping generations structure allows us to disaggregate across generations the associated aggregate welfare gain. Again, the asset price has significant implications for the distribution of welfare across generations. The initial asset owner captures the capital gains resulting from trade. Even under a social planner, trade may reduce future generations’ welfare.

In future research, it would be interesting to examine formally the effect of establishing property rights under autarchy, where we noted that selfish agents have an incentive to use resource policy. This analysis would make it possible to determine the distributional effects of establishing property rights, and the magnitude of the endogenous policy. The distributional effects would certainly depend on how the property rights are established. It would also be interesting to generalize the model by including endogenously changing capital. These extensions would produce a richer analysis of welfare.

### Supplementary Information

Below is the link to the electronic supplementary material.

Supplementary material 1 (pdf 416 KB)

## Proofs and Verification of a Claim

### Proof

Lemma 1 Part (i): Given our assumption that the open economy remains diversified and that both goods are essential in consumption, the mobile factor needs to be indifferent between working in either sector:

9 $$\begin{aligned} w=m^{\prime }(1-L)=(1-\tau )px\gamma . \end{aligned}$$

In the diversified open economy, *p* is exogenous and we have

$$\begin{aligned} \frac{\mathrm{d}L}{\mathrm{d}\tau }= & {} \frac{px\gamma }{m^{\prime \prime }(1-L)}~~<0 \\ \frac{\mathrm{d}L}{\mathrm{d}x}= & {} -\frac{(1-\tau )p\gamma }{m^{\prime \prime }(1-L)}>0 \end{aligned}$$

with the inequality due to decreasing marginal returns in the manufacturing sector.

Current real income in the open economy is $p^{-\alpha }Y=p^{-\alpha }\left( px\gamma L+m(1-L)\right)$. Taking the total derivative with respect to $\tau$ , we find that

$$\begin{aligned} \frac{\mathrm{d}p^{-\alpha }Y}{\mathrm{d}\tau }=p^{-\alpha }\left( px\gamma -m^{\prime }(1-L)\right) \frac{\mathrm{d}L}{\mathrm{d}\tau }=p^{1-\alpha }x\gamma \tau \frac{\mathrm{d}L}{\mathrm{d}\tau } \le 0, \end{aligned}$$

with the second equality following from substituting no arbitrage condition ( 9). Current real income is maximized iff $\tau =0$.

In the closed economy, *p* is endogenous and ensures that goods markets clear. For Cobb–Douglas preferences, the expenditure share of good *R* is $\alpha$, a constant, implying

10 $$\begin{aligned} \frac{\alpha }{1-\alpha }=\frac{pR}{M}=\frac{px\gamma L}{m(1-L)}. \end{aligned}$$

Using Eqs. (9) and (10) and noting that $p\left( 1-\tau \right) \gamma x=w\Longrightarrow pR=\frac{wL}{ \left( 1-\tau \right) }$, equilibrium *L* solves

$$\begin{aligned} \frac{\alpha }{1-\alpha }(1-\tau )m(1-L)=m^{\prime }(1-L)L. \end{aligned}$$

Note that the equilibrium allocation of labor and with it the wage, output in each sector, and manufacturing profits are independent of the stock of the resource, *x*. A computation establishes

11 $$\begin{aligned} \frac{\mathrm{d}L}{\mathrm{d}\tau }= & {} \frac{\alpha }{1-\alpha }m(.)\left[ -m^{\prime }(.)\left( 1+(1-\tau )\frac{\alpha }{1-\alpha }\right) +m^{\prime \prime }(.)L\right] ^{-1}<0 \end{aligned}$$

12 $$\begin{aligned} \frac{\mathrm{d}L}{\mathrm{d}x}= & {} 0. \end{aligned}$$

Using Eq. (10), $p^{-\alpha }Y=\left( \frac{M}{ 1-\alpha }\right) ^{1-\alpha }\left( \frac{R}{\alpha }\right) ^{\alpha }=\left( \frac{m(1-L)}{1-\alpha }\right) ^{1-\alpha }\left( \frac{x\gamma L}{ \alpha }\right) ^{\alpha }$. With the elasticity of current real income with respect to *L*, $\varepsilon _{p^{-\alpha }Y,L}=(1-\alpha )\frac{\mathrm{d}M}{\mathrm{d}L}/ \frac{M}{L}+\alpha \frac{dR}{dL}/\frac{R}{L}=-\alpha (1-\tau )+\alpha =\alpha \tau$, we have

$$\begin{aligned} \frac{\mathrm{d}p^{-\alpha }Y}{\mathrm{d}\tau }=\frac{\mathrm{d}p^{-\alpha }Y}{\mathrm{d}L}\frac{\mathrm{d}L}{\mathrm{d}\tau } =\varepsilon _{p^{-\alpha }Y,L}~\frac{p^{-\alpha }Y}{L}\frac{\mathrm{d}L}{\mathrm{d}\tau } =\alpha \tau \frac{p^{-\alpha }Y}{L}\frac{\mathrm{d}L}{\mathrm{d}\tau }\le 0. \end{aligned}$$

In the closed economy, current real income is maximized if $\tau =0$.

Part (ii): With $\frac{dL}{d\tau }<0$ in the closed and diversified open economy, differentiating equation (4) with respect to $\tau$ establishes for either trade regime,

$$\begin{aligned} \frac{\mathrm{d}x_{t+1}}{\mathrm{d}\tau _{t}}=-\gamma x_{t}\frac{\mathrm{d}L}{\mathrm{d}\tau }>0. \end{aligned}$$

$$\square $$

Proposition 1 Part (iii) is more general than the corresponding result in Karp and Rezai [28]. That paper uses a Cobb–Douglas production function in Manufacturing, and here we use a CES function.

### Proof

Proposition 1 Part (i): In the diversified open economy, current real income equals $p^{-\alpha }Y=p^{-\alpha }\left( px\gamma L+m(1-L)\right)$. A simple computation establishes

$$\begin{aligned} \frac{\mathrm{d}p^{-\alpha }Y}{\mathrm{d}x}=p^{-\alpha }\left( p\gamma L+w\left( \frac{1}{ 1-\tau }-1\right) \frac{\mathrm{d}L}{\mathrm{d}x}\right) >0. \end{aligned}$$

In the closed economy, $\frac{\mathrm{d}L}{\mathrm{d}x}=0\Rightarrow \frac{\mathrm{d}Y}{\mathrm{d}x}=\frac{ \mathrm{d}(m(1-L)+(1-\tau )^{-1}Lm^{\prime }(1-L))}{\mathrm{d}x}=0$. A higher resource stock lowers the relative price of the resource good, $\frac{\mathrm{d}p}{\mathrm{d}x}=-\frac{\alpha }{1-\alpha }\frac{m(1-L)}{x^{2}\gamma L}<0$ from equation (10). It follows

$$\begin{aligned} \frac{\mathrm{d}p^{-\alpha }Y}{\mathrm{d}x}=-\alpha p^{-(1+\alpha )}Y\frac{\mathrm{d}p}{\mathrm{d}x}>0. \end{aligned}$$

Part (ii): In the diversified open economy,

$$\begin{aligned} \frac{\mathrm{d}p^{-\alpha }w}{\mathrm{d}x}= & {} \frac{\mathrm{d}p^{1-\alpha }(1-\tau )x\gamma }{\mathrm{d}x} =p^{1-\alpha }(1-\tau )\gamma >0 \\ \frac{\mathrm{d}p^{-\alpha }\pi }{\mathrm{d}x}= & {} p^{-\alpha }\frac{\mathrm{d}\pi }{\mathrm{d}L^{m}}(-1)\frac{\mathrm{d}L }{\mathrm{d}x}<0 \end{aligned}$$

with $\frac{\mathrm{d}\pi }{\mathrm{d}L^{m}}>0$ as an increase in the mobile factor increases the return to the fixed factor and $\frac{\mathrm{d}L^{m}}{\mathrm{d}L}=-1$.

Part (iii) In the closed economy,

$$\begin{aligned} \frac{\mathrm{d}p^{-\alpha }w}{\mathrm{d}x}= & {} -\alpha p^{-(1+\alpha )}w\frac{\mathrm{d}p}{\mathrm{d}x}>0 \\ \frac{\mathrm{d}p^{-\alpha }\pi }{\mathrm{d}x}= & {} -\alpha p^{-(1+\alpha )}\pi \frac{\mathrm{d}p}{\mathrm{d}x}>0. \end{aligned}$$

$$\square $$

### Proof

(Lemma 2) In the open and closed economy, a tax increases future stocks (Lemma 1): $\frac{ \mathrm{d}x_{t+1}}{\mathrm{d}\tau _{t}}>0$. Under Assumption 1, this increase also increases the resource stock in all future periods: $\frac{ \mathrm{d}x_{t+i}}{\mathrm{d}x_{t+1}}>0$ for $i>1$. Higher future levels of the resource stock decreases future real returns to capital in the diversified open economy (Proposition 1.ii) and increases future real returns to capital in the closed economy (Proposition 1.iii). Since the real wealth, $p_{t}^{-\alpha }\sigma _{t}$, is the present value of future real returns (Eq. 2), a higher tax increases real wealth in the closed economy and decreases real wealth in the diversified open economy. $\square$

### Proof

(Proposition 2) (Part i) In both the open and closed economies, a zero tax maximizes real national income; by continuity of derivatives, a small tax has a zero first-order effect on real national income. Moreover, increasing $\left| \tau \right|$ lowers real national income.

In the diversified open economy, a tax lowers real wealth (Lemma 2). Therefore, a negative tax (a subsidy) dominates $\tau \ge 0$. Further reductions in the tax (i.e., increases in the subsidy) create a negative first-order effect on national income and a positive first-order effect on wealth. As $\tau \rightarrow -\infty$, the manufacturing sector becomes vanishingly small, and resource extraction approaches the upper bound (where $L\rightarrow 1$). Thus, the sum of national income and wealth is bounded as $\tau \rightarrow -\infty$. The sum therefore reaches a maximum at $\tau <0$.

The closed economy reverses this logic. Here, a subsidy reduces both real national income and real wealth, so $\tau =0$ dominates $\tau <0$. A small tax has a zero first-order effect on real income and a positive first-order effect on real wealth, so a small positive tax dominates $\tau =0$. As $\tau \rightarrow 1$, the economy specializes in manufacturing, causing current utility (real national income) to approach 0 and real wealth to approach an upper bound. Thus, welfare is bounded, and maximized at a positive tax in the closed economy.

Part ii follows from the boundary condition that the price of capital at the end of the final period is 0. Therefore, the objective in the final period is to maximize national income, which requires a zero tax. $\square$

*Conditions for Assumption 1.i to hold* Using $\frac{\mathrm{d}x_{t+1}}{\mathrm{d}L_{t}}<0$, we restate the monotonicity assumption, $\frac{\mathrm{d}x_{t+i}}{\mathrm{d}L_{t}}<0$ for$~i=1,2,\ldots ,H$, as $\frac{\mathrm{d}x_{t+1+i}}{\mathrm{d}x_{t+1}} >0$. This inequality holds if and only if $\frac{\mathrm{d}x_{t+i+2}}{\mathrm{d}x_{t+i+1}}>0$. In the closed economy, for $\eta =1$ and $\tau =0$, the inequality is satisfied [28] for

$$\begin{aligned} 1<\varsigma <2\text { with }\varsigma \equiv r+\frac{\beta \left( 1-\alpha \right) +\alpha \left( 1-\gamma \right) }{\beta \left( 1-\alpha \right) +\alpha }\text {,} \end{aligned}$$

provided that the initial condition is “not too large” (in particular, it lies below the level at which $\bar{r}(x_{t},0)=-1$).

In the open economy, also for $\eta =1$ and $\tau =0$, the diversified equilibrium condition $\beta \left( 1-L\right) ^{\beta -1}=P\gamma x$ implies $L\left( x\right) =1-\left( P\gamma x/\beta \right) ^{\frac{1}{\beta -1}}$. Define

$$\begin{aligned} J=\left\{ x\left| 1+\frac{r}{C}\left( 1-2x\right) -\frac{\gamma }{ 1-\beta }\left( 1-\beta L\left( x\right) \right) >0\right. \right\} . \end{aligned}$$

Using Eq. (4), we have

$$\begin{aligned} \frac{\mathrm{d}~x_{s+1}}{\mathrm{d}~x_{s}}\ge 0\Leftrightarrow x_{s}\in J. \end{aligned}$$

Assumption 1.i is satisfied if and only if $x_{0}\in J$ and $x_{\infty }\in J$. Using examples, it is easy to confirm that for some parameters, *J* is nonempty and $x_{\infty }\in J$.

## Footnotes

1 Some resources, particularly fisheries, are managed using individually transferable quotas (ITQs), guaranteeing quota owners a share of a stream of aggregate quotas chosen by current and future regulators. In our model, the lack of de jure property rights prevents one generation from selling a resource-based asset to its successor.

2 Previous OLG models with natural resources or environmental applications include Kemp and Long [30], Howarth and Norgaard [23], John and Pecchenino [24], Bovenberg and Heijdra [6], and Laurent-Lucchetti and Leach [34]. These papers show how a benevolent planner who can tax and issue secure bonds can lead to Pareto improvements. Karp and Rezai [28] study equilibrium policy determination for a generic resource, using a less general production function. Karp et al. [27] study a one-commodity model with endogenous investment. Both of these papers consider a closed economy. The focus of the current paper is on the effect of the trade regime, so we need to consider both open and closed economies.

3 Trade changes the incidence of taxes because commodity prices are endogenous in the closed economy, but fixed for a small open economy. With fixed commodity prices, real returns to a factor are proportional to nominal returns: an agent’s real income (utility) from renting a factor of production rises if and only if the nominal return to that factor increases. Under autarchy, the commodity price varies with the relative supply of the commodity; a policy change can cause the real and nominal returns to a factor to change in opposite directions.

4 In both a Nash bargaining model with transfers and in a probabilistic voting model [35, 45], an equilibrium maximizes the weighted sum of agents’ welfare. Our results do not depend on these micro-foundations. See also the discussion in Sect. 5. There are, of course, other types of political economy equilibria, as in Battaglini and Coate [3], who consider a dynamic game among infinitely lived agents.

5 We thank an anonymous referee who sketched the reasoning that we develop in the rest of this section.

6 In a competitive equilibrium with endogenous prices, the shadow value of the resource measures the actual marginal social value of an additional unit of stock. Future consumers obtain some of that benefit via the lower resource price. Selfish currently living people in our model place no value on this increase in future welfare.

7 We cannot confirm this conjecture without considerably more analysis, well beyond the scope of this paper. The difficulty is that with property rights, a resource subsidy also affects the value of the fixed capital stock, $\sigma$. Our *formal* analysis considers only the effect of trade liberalization under open access.

8 Appendix B.5 of ESM considers the general case when the fraction of the tax payments or the subsidy cost that young agents receive or pay is endogenous.

9 Appendix B of ESM contains further details. For infinite horizon models, there is a generic multiplicity of equilibria, and we think that our model is no exception. There is often a unique “limit equilibrium,” obtained by taking the limit of the finite horizon game as $H\rightarrow \infty$. The numerical algorithm mimics this procedure and produces, for all of our experiments, a unique equilibrium.

10 With six distinct policy scenarios, we have 30 possible pairs of policies. Because welfare effects differ for the young and the old, we have 60 possible welfare comparisons. We consider only the most interesting of these possibilities.

11 In both regimes, a higher stock draws labor into the resource sector, lowering the nominal return to capital. The reduction in the commodity price due to the higher stock offsets this change, increasing the real return to capital in the closed economy. This offsetting price effect does not arise in the open economy.
