# Supplementary material for: Trade and Resource Sustainability with Asset Markets
Source: Dyn Games Appl. 2021 Nov 2;12(3):929–53. doi: 10.1007/s13235-021-00400-4 (PMC9338156; doi:10.1007/s13235-021-00400-4)
Supplement: Supplementary file 1 — Supplementary material 1 (pdf 416 KB) [file 13235_2021_400_MOESM1_ESM.pdf]

B Supplementary Appendices (For Online Publication)

Supplementary Appendix B.1 contains details of the numerical algorithm, which relies on standard methods of function approximation. Supplementary Appendix B.2 describes our sensitivity studies, mentioned in Section 5. Supplementary Appendix B.3 compares the effect of moving from BAU to the social planner solution, holding fixed the trade regime. We think that the comparisons in Supplementary Appendices 5.3 and B.3 are of general interest, but, apart from brief comments, we have removed it from the paper in the interest of brevity and in order to focus on our principal research questions. Supplementary Appendix B.4 presents an analytical solution to the static equilibrium for the special case of unit elasticity in production, the case used in the numerical simulations. In Supplementary Appendix B.5 we allow for a flexible sharing rule of tax revenue / subsidy cost T across generations.

B.1 MPE solution algorithm

Agents at time t take the functions $\Upsilon(x_{t+1})$ and $\sigma(x_{t+1})$ as given, but they are endogenous to the problem. We solve $\max_\tau W(x,\tau)$ using a standard dynamic programming algorithm. An arbitrary policy function, $\Upsilon^k(x_t)$, induces the real asset price, $\sigma^k(x_t,\tau_t)$, given by equation (6); the superscript k denotes the functional dependence of $\sigma^k(x_t,\tau_t)$ on the function $\Upsilon^k(x_t)$. Replacing $\sigma(x_t,\tau_t)$ with $\sigma^k(x_t,\tau_t)$ and W_t with W_t^k in the maximand, we denote

$$\Upsilon^{k+1}(x) = \arg\max_\tau W^k(x,\tau).$$

This relation is a mapping from Υ^k to Υ^{k+1}. An equilibrium Υ is a fixed point to this mapping, which we approximate using the collocation method and Chebyshev polynomials (Judd, 1998; Miranda and Fackler, 2002).

Infinite horizon games of this genus have multiple equilibria, arising from an "incomplete transversality condition". Tsutsui and Mino (1990) discuss this issue in the context of a differential game. Karp (2007) shows that it also occurs under nonconstant discounting, and Ekeland and Lazrak (2010) show that it holds in an OLG model, which is a special case of nonconstant discounting. The multiplicity occurs because the equilibrium conditions, evaluated in the steady state, do not determine agents' beliefs about how other

agents would respond to a deviation that drives the state away from the steady state. The infinite horizon version of our game has this characteristic, and therefore "very likely" has multiple equilibria.

One equilibrium refinement uses the limit of the finite horizon model, as the horizon goes to infinity. The equilibrium to each of the games (indexed by the length of the horizon) in this sequence may or may not be unique. This determination must be made on a case by case basis. For problems as complex as ours, there seems no alternative but to rely on numerical methods to assess uniqueness. Our method for numerically approximating the equilibrium to the infinite horizon game is formally identical to finding the equilibrium to a game whose horizon is long enough that increases in the horizon have negligible effect on the decision rule early in the program. By showing numerically that the equilibrium does not depend on the initial guess for the equilibrium control rule and asset price functions, we provide evidence that our refinement (take the limit of a finite horizon game) is unique. To the best of our knowledge this is the state of the art in this literature. Fujii and Karp (2008) discuss this issue in much more detail and provide an example in which the numerical algorithm identifies the analytic limit of the finite horizon model.

The open loop and Markov perfect equilibria are studied in the same manner: in each case by taking the limit of a finite horizon model.

To simplify notation, we introduce a new function, the value of the asset in units of utility (rather than in units of the numeraire good):

$$\bar{\sigma}\left(x_{t+1}, \Upsilon\left(x_{t+1}\right)\right) = p^{-\alpha}\left(x_{t+1}, \Upsilon\left(x_{t+1}\right)\right)\sigma\left(x_{t+1}, \Upsilon\left(x_{t+1}\right)\right).$$

We approximate $\Upsilon\left(x_{t+1}\right)$ and $\bar{\sigma}\left(x_{t+1}, \Upsilon\left(x_{t+1}\right)\right) \equiv \Phi\left(x_{t+1}\right)$ as polynomials in x_{t+1}, and find coefficients of those polynomials so that the solution to

$$\begin{gathered}\max_{\tau_t} P^{-\alpha}\left(x_t, \tau_t\right) Y\left(x_t, \tau_t\right) + \\ \tfrac{1}{1+\rho}\left\{P^{-\alpha}(x_{t+1}, \Upsilon\left(x_{t+1}\right))\pi\left(x_{t+1}, \Upsilon\left(x_{t+1}\right)\right) + \Phi\left(x_{t+1}\right))\right\}\end{gathered}$$

subject to equation (4) approximately equals $\Upsilon\left(x_t\right)$. We use Chebyshev polynomials and Chebyshev nodes. At each node, the recursion defining $\bar{\sigma}\left(x_{t+1}, \Upsilon\left(x_{t+1}\right)\right)$,

$$\Phi\left(x_t\right) = \frac{1}{1+\rho}\left\{p^{-\alpha}(x_{t+1}, \Upsilon\left(x_{t+1}\right))\pi\left(x_{t+1}, \Upsilon\left(x_{t+1}\right)\right) + \Phi\left(x_{t+1}\right))\right\} \tag{13}$$

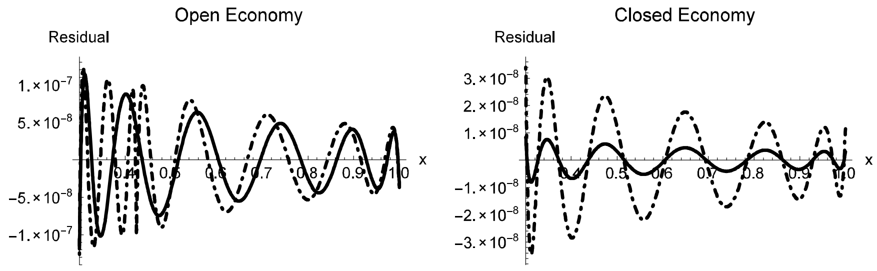

Figure 5: Approximation error for asset price function (LHS-RHS of (13)): the MPE (solid) and the social planner's (dot-dashed) problems, the open economy (left) and closed economy (right).

and the optimality condition

$$\frac{d}{d\tau_t}\left[P^{-\alpha}\left(x_t,\tau_t\right)Y\left(\tau_t\right)+\frac{1}{1+\rho}\Omega\right]=0, \tag{14}$$

with

$$\Omega \equiv \left\{P^{-\alpha}(x_{t+1},\Upsilon\left(x_{t+1}\right))\pi\left(\Upsilon\left(x_{t+1}\right)\right)+\Phi\left(x_{t+1}\right)\right)\right\}$$

must be satisfied.

Starting with an initial guess for the coefficients of the approximations of $\Phi(\cdot)$ and $\Upsilon(\cdot)$, we evaluate the right side of equation (13) for at each node. Using these function values, we obtain new coefficient values for the approximation of $\Phi(\cdot)$. We then use the optimality condition (14) to find the values of $\Upsilon(\cdot)$ at each node; we use those values to update the coefficients for the approximation of $\Upsilon(\cdot)$. We repeat this iteration until the coefficients' difference between iterations, relative to the estimated value of the coefficient, falls below 10^{-6}. See chapter 6 of Miranda and Fackler (2002) for details.

The social planner uses a prohibitive tax in the open economy when the stock is low. We approximated the point of specialization through numerical experiments and at first limited the approximation space to the range of diversified production $x \in [0.4246, 1]$. Under a prohibitive tax, this set also contains all x_{t+1} for $x_t \in [0.3, 0.4146)$. Given the approximations of $\Phi(\cdot)$ and $\Upsilon(\cdot)$ for the set of diversified production, one can use recursion (13) to approximate $\Phi(\cdot)$ for the range of specialized production. As $\Phi(\cdot)$ might not

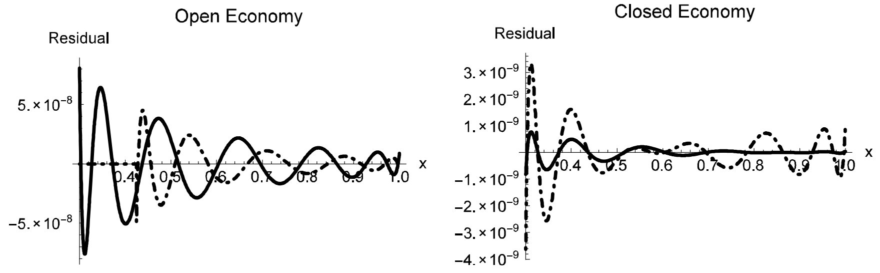

Figure 6: Approximation error for policy function (LHS-RHS of (14)): the MPE (solid) and the social planner's (dot-dashed) problems, the open economy (left) and closed economy (right).

be smooth at $x = 0.4246$, we used separate polynomials for the ranges of diversified and specialized production.

Figures 5 and 6 graph the differences (the "residuals") between the right and left sides of equations (13) and (14), respectively. These residuals equal 0 at the nodes, because we set both the degree of the polynomial and the number of nodes equal to n. We choose $n = 12$, yielding residuals that are at least 6 orders of magnitudes below the solution values on the $[0.3, 1]$ interval. In the case of a social planner in the open economy, we chose $n = 10$ for $x \in [0.4246, 1]$ interval and $n = 6$ for $x \in [0.3, 0.4246)$.

Given the assumption of Cobb Douglas production in the numerical simulations, we can utilize equilibrium expressions presented in appendix B.4.

B.2 Numerical sensitivity

Proposition 2 establishes that (under Assumption 1), except for the last period, the equilibrium policy is a sequence of subsidies under trade and of taxes in the closed economy. The numerical results reported in the text show that these qualitative differences also hold in the MPE. To confirm that our numerical results (a sequence of subsidies in the open economy and of taxes in the closed economy) are not an artifact of one particular parameter set, we conduct extensive parameter sensitivity analysis. We define the following values for the model's parameters (with bold numbers indicating the baseline value used in the text), and determine the corresponding equilibrium policy

for each combination of parameters that satisfy certain restrictions described below.

$$\alpha = \{0.1, 0.3, \mathbf{0.5}, 0.7, 0.9\}$$
$$\rho = \{0.1,\ \mathbf{0.41},\ 0.7\}$$
$$\beta = \{0.4,\ \mathbf{0.5},\ 0.6\}$$
$$r = \{0.1,\ 0.5,\ \mathbf{0.68},\ 0.9,\ 1.1\}$$
$$\gamma = \{0.1,\ 0.3,\ \mathbf{0.513},\ 0.7,\ 0.9\}$$
$$P = \{1,\ 2,\ 3,\ \mathbf{3.377},\ 4,\ 6,\ 9\}$$

In the sensitivity runs we reduce the number of collocation points to 8.

For both the open and closed economy, we include only parameters that, under BAU, lead to monotonic adjustment (the BAU x_{t+1} is an increasing function of x_t, and crosses the 45° line with slope less than 1). For the closed economy the state space is $x \in [0.05, 1]$. For the open economy, the state space is the subspace of the closed economy on which the open BAU economy remains diversified. Under specialization, equilibrium policy is indeterminate, so we do not consider that case.

Given that the MPE only involves expressions in utility, the value of α has no effect on the open economy equilibrium ($P^{-\alpha}$ reduces to a scaling parameter). We hold α constant at the baseline value, $\alpha = 0.5$, and begin with $3 \times 3 \times 5 \times 5 \times 7 = 1575$ combinations of parameter values. Of these, 915 combinations lead to monotonic BAU growth paths. At $x = 0.9$, there are 813 parameter combinations that imply both monotonic BAU paths and diversification; at $x = 0.1$, there are 120 such parameter combinations (see legend of left panel in Figure 7).

For the closed economy, the relative commodity price is endogenous, and depends on α. We begin with $5 \times 3 \times 3 \times 5 \times 5 = 1125$ parameter combinations. Of these, 1065 parameter combinations lead to monotonic BAU adjustment; 780 combinations lead to both monotonic BAU adjustment and BAU steady states in the interval $[0.05, 0.95]$.

Figure 7 shows box plots for the distribution of the equilibrium policy, at different values of x. For all parameter combinations included in these plots, the policy is a subsidy for the open economy and a tax for the closed economy. The numbers at the right of each figure show the number of parameter combinations used for each value of x; this number increases with x in the open economy (as production becomes diversified in more cases) and is constant in the closed economy. Each box contains the middle quartiles (Q2 and Q3, 25%-75%) while the lower and upper whiskers give Q1 (0%-25%) and Q4 (75%-100%). The white line in the box shows the median subsidy/tax

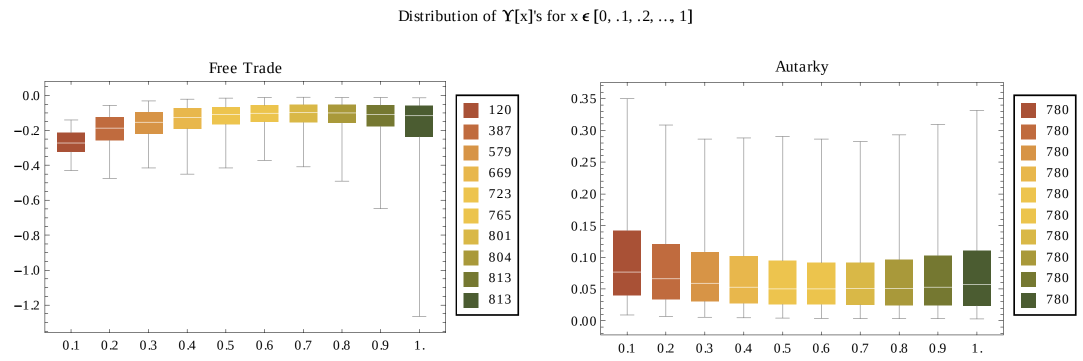

Figure 7: Parameter sensitivity of MPE: box-whisker plots of distribution of policy, τ, as a function of the resource stock, x, for free trade (left panel) and autarchy (right panel)

for a given value of x. The sensitivity results summarized in Figure 7 confirm that the equilibrium in the open economy involves a subsidy, and the equilibrium in the closed economy involves a tax for a large parameter space. In summary, we do not find any parameter combinations that overturn these results; but we did not consider combinations that violate the monotonicity and diversification (under BAU) restrictions.

B.3 The social planner

The dot-dash graphs in Figure 1 show the equilibrium policy functions, asset prices, and state transitions for the social planner. In both the open and closed economies, the planner uses a resource tax; in both trade regimes, the equilibrium stock and tax trajectories are higher under the social planner compared with both BAU and MPE. Under trade for $x < 0.42$, the social planner uses a prohibitive tax, allowing the resource to grow as fast as possible. Under diversified production, the tax remains close to its steady state level, $\tau_\infty = 0.32$, at $x_\infty = 0.61$. The closed economy steady state tax is higher, $\tau_\infty = 0.36$, but the steady state stock is lower, $x_\infty = 0.58$. The social planner achieves greater protection of the resource at a lower tax, in the open compared to closed economy.

For $t \geq 1$ Figures 8 shows welfare of the young generation under the SP relative to BAU. For $t = 0$ the figure shows the aggregate lifetime welfare

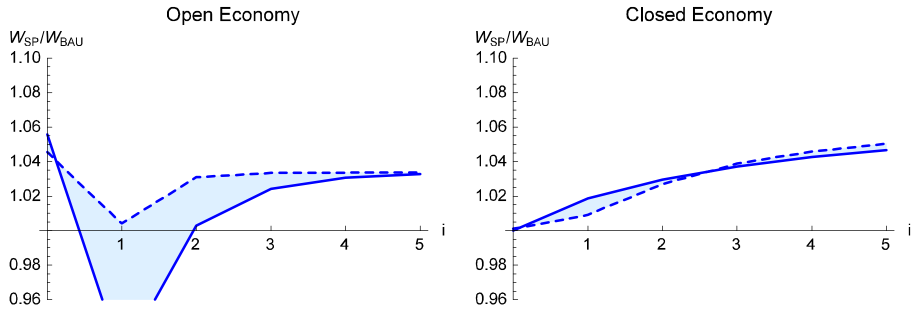

Figure 8: Welfare under the social planner relative to BAU with the initial resource stock $x_0 = 0.5$ (dashed) and $x_0 = 0.9$ (solid) for open economy (left) and closed economy (right).

ratio of currently living agents.

In Ramsey models, optimal resource policy requires that currently living agents sacrifice to benefit future agents. Our social planner solves the standard intertemporal problem (8), so it is no surprise that her program lowers aggregate period 0 *utility*. The planner's policy function induces a trajectory of welfare for the old and young agents. The planner's program in the closed economy leads to a slight increase in period 0 aggregate lifetime *welfare* for initial conditions $x_0 < 0.91$ (Figure 8) and a small loss at larger stocks. The planner's program increases the asset price (Figure 1, panels *c* and *d*), and the old generation alive in period 0 obtains these capital gains.

Those alive in the initial period have a more pronounced policy-induced welfare increase in the open economy, compared to the closed economy. This difference arises because the socially optimal policy creates a larger increase in the asset price in the open economy, compared to the closed economy. Without these capital gains, the initial generations suffer large losses in welfare under the social planner (compared to BAU), in the open economy. In both open and closed economies, the planner's intervention increases the steady state level of welfare, because intervention increases the steady state resource stock. In the closed economy, the planner raises welfare for all generations, if $x_0 < 0.91$. In the open economy, the planner reduces intermediate generations' welfare if the initial resource stock is high. Those generations would not have suffered much from a low stock under BAU, but they have a

lower real wage when the planner taxes the resource.

B.4 Competitive Equilibrium for unit elasticity

Here we specialize to unit elasticity, $\eta = 1$, so the production function $m(.)$ is Cobb Douglas. With this specialization we obtain closed form expressions for endogenous functions. We show that, conditional on the resource stock, a resource tax in the closed economy increases the young agent's welfare; in the open economy, a resource subsidy increases the young agent's welfare.

Factor returns in manufacturing equal:

$$\begin{aligned}\pi_t &= m_K(L_t^m, 1) = (1-\beta)\left(L_t^m\right)^{\beta} \\ w_t^m &= m_{L^m}(L_t^m, 1) = \beta\left(L_t^m\right)^{\beta-1}\end{aligned}$$

In the resource sector, the mobile factor earns:

$$w_t = p_t(1-\tau_t)\gamma x_t$$

Goods and factor markets clear. Full employment of the mobile factor requires $L_t^m = 1 - L_t$. We assume $\beta < 1$, so rents are positive in sector M and capital is fully employed. No-arbitrage requires that the mobile factor is indifferent to working in either sector. The Cobb Douglas production function also implies that some labor is always used in the manufacturing sector: $L_t^m > 0$. Returns to working in the resource sector can, however, be too low to attract the mobile sector:

$$w_t \le w_t^m \qquad \perp \qquad L_t \ge 0.$$

Agents' period utility function is Cobb Douglas: $u(c_{R,t},\ c_{M,t}) = \frac{1}{\mu}c_{R,t}^{\alpha}c_{M,t}^{1-\alpha}$, with $0 < \alpha < 1$ the constant budget share for the resource-intensive good; the scaling parameter is $\mu = \alpha^{\alpha}(1-\alpha)^{1-\alpha}$. Cobb Douglas preferences imply that both goods are essential and both sectors operate in the closed economy. With e_t equal to expenditures, an agent's single period indirect utility is

$$v(e_t, p_t) = \frac{1}{\mu}\left(\frac{\alpha e_t}{p_t}\right)^{\alpha}\left(\frac{(1-\alpha)\,e_t}{1}\right)^{1-\alpha} = p_t^{-\alpha}e_t.$$

With identical homothetic preferences, the share of income devoted to each good is independent of both the level and distribution of income.

In the open economy, p_t is exogenous and trade balances. In the closed economy, the relative price, p_t, adjusts to ensure that domestically produced supply equals domestic demand:

$$\frac{p_t R_t}{M_t} = \frac{\alpha}{1-\alpha}.$$

These equilibrium conditions for the labor and product markets lead to the following expressions for the values of L_t, w_t, and p_t:

Closed Economy	Open Economy (diversified)
$L_t = \frac{1-\tau_t}{\frac{1-\alpha}{\alpha}\beta+1-\tau_t}$ $w_t = \beta\left(1+\frac{1-\tau_t}{\frac{1-\alpha}{\alpha}\beta}\right)^{1-\beta}$	$L_t = 1-\left(\frac{p_t(1-\tau_t)\gamma x_t}{\beta}\right)^{-\frac{1}{1-\beta}}$ $w_t = p_t(1-\tau_t)\gamma x_t$
$p_t = \frac{\beta\left(1+\frac{1-\tau_t}{\frac{1-\alpha}{\alpha}\beta}\right)^{1-\beta}}{(1-\tau_t)\gamma x_t}$	$p_t = P$ given.

Under trade, for $p \leq \frac{\beta}{(1-\tau)\gamma x}$, the economy specializes in sector M: $L_t = 0$ and $w_t = \beta$.

The period equilibrium depends on the resource stock, the asset price, and the tax: x_t, σ_t and τ_t. A competitive equilibrium at t, conditional on x_t and $\{\tau_{t+h}\}_{h=0}^{H-t}$, is a sequence of resource stocks and asset prices, $\{x_{t+h}, \sigma_{t+h}\}_{h=0}^{H-t}$, satisfying the asset price equation (2), the resource transition equation (4), and the static equilibrium.

Lifetime welfare of the young agent is $p^{-\alpha}(w+T)$. In the closed economy, this equals:

$$p^{-\alpha}(w+T)\Big|_{c.e.} = \left(\frac{\beta\left(1+\frac{\alpha(1-\tau)}{(\alpha)\beta}\right)^{1-\beta}}{\gamma(1-\tau)x}\right)^{-\alpha} \times \left(\beta\left(1+\frac{\alpha(1-\tau)}{(1-\alpha)\beta}\right)^{1-\beta} - \frac{\alpha\tau\left(1+\frac{\alpha(1-\tau)}{(1-\alpha)\beta}\right)^{-\beta}}{1-\alpha}\right)$$

and the effect of a change in the policy τ equals

$$\frac{\partial(p^{-\alpha}(w+T))}{\partial\tau}\Big|_{c.e.} = -p^{-\alpha}(w+T)\frac{(1-\alpha)\beta}{(1-\tau)^2}L\,\tau \qquad (15)$$

In the closed economy, lifetime welfare of the young decreases in the policy, provided that the policy is a tax (i.e. τ and therefore T positive).

In the open economy, we have:

$$p^{-\alpha}(w+T)\Big|_{o.e.} = p^{1-\alpha}\left(1-\tau\left(\frac{p(1-\tau)\gamma x}{\beta}\right)^{\frac{1}{\beta-1}}\right)\gamma x$$

and the effect of a change in the policy τ equals

$$\frac{\partial(p^{-\alpha}(w+T))}{\partial\tau}\Big|_{o.e.} = -p^{1-\alpha}(1-L)x\gamma\left(1+\frac{\tau}{(1-\beta)(1-\tau)}\right) \tag{16}$$

In the open economy, where the equilibrium policy is a subsidy ($\tau < 0$, so $T < 0$) lifetime welfare of the young increases in the policy, provided that the subsidy is small enough to satisfy $1 - 1/\beta < \tau < 0$.

B.5 General tax share χ

In the interest of simplicity, the text assumes that the young agents receive all of tax revenue, or pay all of the fiscal cost of a subsidy, $T_t = \tau_t p_t \gamma x_t L_t$. Here we allow for a flexible and endogenous sharing rule, giving the fraction $\chi_t \in [0,1]$ to the young and $1-\chi_t$ to the old in period t. If the tax is negative (a subsidy), $T_t < 0$ and the policy has a fiscal cost; χ_t determines the generations' share of this cost. In the main text $\chi_t = 1$.

With general χ_t, the young agent's decision problem changes to

$$\max_{s_t} p_t^{-\alpha}\left(w_t + \chi_t T_t - s_t\sigma_t\right) + \frac{1}{1+\rho} p_{t+1}^{-\alpha}\left(\left(1-\chi_{t+1}\right)T_{t+1} + s_t\left(\pi_{t+1} + s_{t+1}\sigma_{t+1}\right)\right).$$

This generalization does not alter the optimal saving decision (1) because of the infinite intertemporal elasticity of substitution.

Equilibrium welfare for the young and old generations, W_t^y and W_t^o, becomes:

$$W_t^y = p_t^{-\alpha}\left[w_t + \chi_t T_t\right] + \tfrac{1}{1+\rho} p_{t+1}^{-\alpha}\left[\left(1-\chi_{t+1}\right)T_{t+1}\right]$$
$$W_t^o = p_t^{-\alpha}\left[\pi_t + (1-\chi_t)T_t + \sigma_t\right].$$

With general χ_t, agents receive tax revenue or pay the cost of the subsidy in the next period, so the open loop tax solves

$$\tau_t = \arg\max_{\tau}\left(p_t^{-\alpha}Y_t + p_t^{-\alpha}\sigma_t + \frac{1}{1+\rho}\left(1-\chi_{t+1}\right)p_{t+1}^{-\alpha}R_{t+1}\right).$$

Lemma 2 and Proposition 2 remain unaffected by allowing $0 \le \chi_t \le 1$. In the closed economy, the equilibrium tax increases next period's resource

stock, lowering p_{t+1} and increasing R_{t+1}. Next period's real tax revenue increases in today's tax, given future resource policy. In the open economy, similar reasoning applies: Today's subsidy lowers next period's resource stock and the total cost of the subsidy. Generalizing χ_t increases the level of the tax (subsidy) in the closed (open) economy.

In determining the MPE we decouple the problem of finding the equilibrium sharing rule from the problem of finding the equilibrium tax policy. Suppose that the political objective function assigns weight 1 to the old generation's welfare, and weight $1+\delta$ to the young generation's welfare, with constant δ. In this setting, the political preference function with general share χ_t is

$$\tilde{W}_t \equiv W_t^o + (1+\delta) W_t^y = p_t^{-\alpha} \left[Y_t + \sigma_t + \delta \left(w_t + \chi_t T_t \right) \right] + \frac{1+\delta}{1+\rho} \left[p_{t+1}^{-\alpha} \left(1 - \chi_{t+1} \right) T_{t+1} \right].$$

The constraint $\chi_t \in [0,1]$ and the linearity of $\tilde{W}_t$ in χ_t, produce the optimal revenue split:

$$\chi_t^* = \arg\max_{\chi_t} \tilde{W}_t = \left\{ \begin{array}{c} 0 \\ \text{indeterminate} \\ 1 \end{array} \right\} \text{ if } \left\{ \begin{array}{c} \delta T_t < 0 \\ \delta T_t = 0 \\ \delta T_t > 0 \end{array} \right\}. \tag{17}$$

Tax revenue, T_t, has the same sign as τ_t. We assume that $\delta \neq 0$, and $|\delta|$ is small.

The assumption $\delta \neq 0$ eliminates the indeterminate case in equation (17), because in equilibrium $T_t \neq 0$. We numerically confirm the hypothesis that, conditional on the trade regime and on $\chi = 0$ or $\chi = 1$, the equilibrium T_t does not change signs. This hypothesis allows us to obtain the tax rule for each trade regime and for both $\chi = 0$ and $\chi = 1$. We find that for both values of χ, and for all x, the equilibrium policy is always a tax in the closed economy and always a subsidy in the open economy, as in the open loop setting under Assumption 1. We then use equation (17) to determine the sign of δ corresponding to a particular value of χ. For example, in the open economy with $\delta > 0$, the equilibrium sharing rule is $\chi^* = 0$: the young pay none of the fiscal cost of the subsidy.

The assumption that $|\delta|$ is small means that agents choose the current tax or subsidy to increase the aggregate lifetime welfare of those currently alive, not to transfer income from one currently living generation to the other; the assumption allows us to replace the political preference function $\tilde{W}_t$ with its

approximation, $W_t = W_t^o + W_t^y$, to compute the equilibrium tax, conditional on $\chi = 0$ or $\chi = 1$. For $\delta \approx 0$, χ_t has a negligible effect on the optimal tax, because χ_t transfers income between two generations with approximately the same weight in the political preference function. In contrast, even for $\delta \approx 0$, χ_{t+1} has a non-negligible effect on the optimal tax; χ_{t+1} determines a transfer between the current young and the next period young, who have zero weight in the political preference function.

Figure 9 reproduces Figure 1 and includes simulations for $\chi = 0$ (dashed, blue). Equilibrium policy function (panels *a* and *b*) and asset prices (panels *c* and *d*) are insensitive to the value of χ in the closed economy, and moderately sensitive to χ in the open economy. In the open economy, where the equilibrium is a subsidy, equation (17) shows that $\chi = 0$ corresponds to $\delta > 0$: the old bear the fiscal cost of the policy. Changing from $\delta < 0$ to $\delta > 0$, i.e. giving the young generation greater weight in the preference function, slightly decreases the resource tax in the closed economy, and increases the resource subsidy in the open economy: greater political weight on the young harms the resource under both trade regimes.

Figure 10 reproduces Figure 4 and includes simulations for $\chi = 0$ for the MPE and SP scenarios (under BAU there are no taxes $\tau = 0$). In the open economy the MPE policy is a subsidy which creates costs while the policy is a tax, which generates revenue, in the closed economy. This difference lowers the welfare gains from free trade of the generation receiving the policy benefits / costs. Shifting these from the young (the baseline case of the main text) to the old generations, i.e. changing χ from 1 to 0, increases the welfare gains of shifting to trade for the young and lowers those of the old generation for all values of the resource stock. Under the social planner, the policy is always a tax. The tax rate in the open economy is still below that of the closed economy and welfare from tax revenues is higher in the closed economy for most values of the stock. Hence, both generations have higher gains from free trade when they do not receive any tax revenue. When they receive tax revenues, those welfare gains drop due to the decrease in revenues they receive.

References

Ekeland, I. and A. Lazrak (2010): "The golden rule when preferences are time inconsistent," *Mathematics and Financial Economics*, 4, 29–55.

Fujii, T. and L. Karp (2008): "Numerical analysis of non-constant pure rate of time preference: a model of climate policy," *Journal of Environmental Economics and Management*, 56, 83–101.

Judd, K. (1998): *Numerical Methods in Economics*, Cambridge, Massachusetts: MIT Press.

Karp, L. (2007): "Non-constant discounting in continuous time," *Journal of Economic Theory*, 132, 557–568.

Miranda, M. J. and P. L. Fackler (2002): *Applied Computational Economics and Finance*, Cambridge, Massachusetts: MIT Press.

Tsutsui, S. and K. Mino (1990): "Nonlinear strategies in dynamic duopolisitc competition with sticky prices," *Journal of Economic Theory*, 52, 136–161.

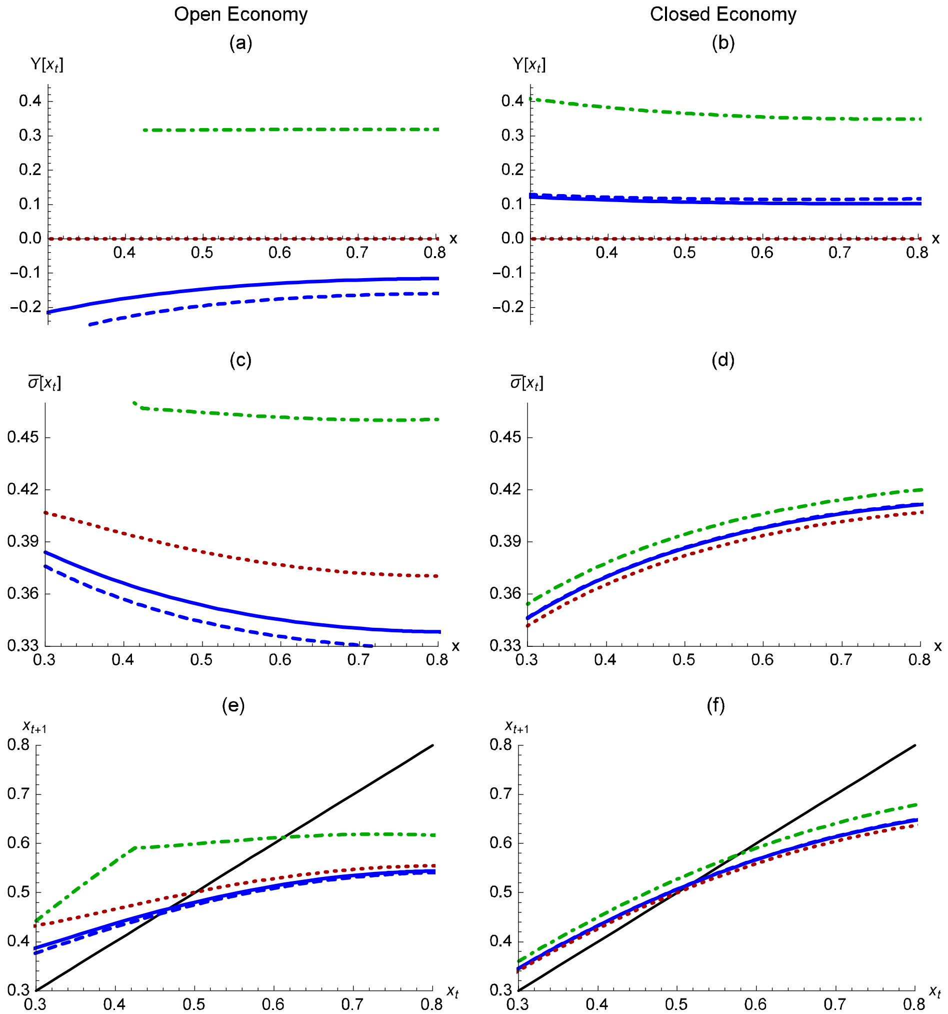

Figure 9: Legend: open economy (left panels) and closed economy (right panels); MPE with $\chi = 1$ (solid blue) and $\chi = 0$ (dashed blue), BAU (dotted red) and social planner (dot-dashed green); equilibrium policy functions (top), equilibrium real wealth (middle), and equilibrium stock transition relation (bottom).

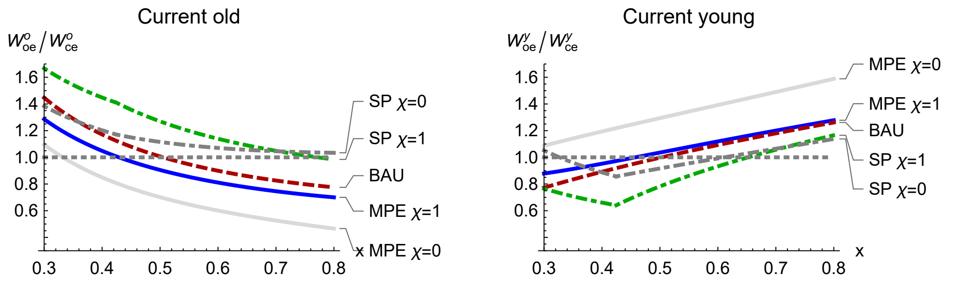

Figure 10: Lifetime welfare of the current generations in the open economy (oe) relative to the closed economy (ce) under MPE, BAU, and SP for $\chi = 0$ or 1 where applicable.
